# Spectral Detection of Nanophase Iron Minerals Produced by Fe(III)-Reducing Hyperthermophilic Crenarchaea

**DOI:** 10.1089/ast.2022.0042

**Published:** 2022-12-30

**Authors:** Srishti Kashyap, Elizabeth C. Sklute, Peng Wang, Thomas J. Tague, M. Darby Dyar, James F. Holden

**Affiliations:** ^1^ Department of Microbiology, University of Massachusetts, Amherst, Massachusetts, USA.; ^2^ Planetary Science Institute, Tucson, Arizona, USA.; ^3^ Bruker Optics, Inc., Billerica, Massachusetts, USA.; ^4^ Department of Astronomy, Mount Holyoke College, South Hadley, Massachusetts, USA.

**Keywords:** Hyperthermophile, Archaea, Iron oxides, Magnetite, Biomineralization

## Abstract

Mineral transformations by two hyperthermophilic Fe(III)-reducing crenarchaea, *Pyrodictium delaneyi* and *Pyrobaculum islandicum*, were examined using synthetic nanophase ferrihydrite, lepidocrocite, and akaganeite separately as terminal electron acceptors and compared with abiotic mineral transformations under similar conditions. Spectral analyses using visible–near-infrared, Fourier-transform infrared attenuated total reflectance (FTIR-ATR), Raman, and Mössbauer spectroscopies were complementary and revealed formation of various biomineral assemblages distinguishable from abiotic phases. The most extensive biogenic mineral transformation occurred with ferrihydrite, which formed primarily magnetite with spectral features similar to biomagnetite relative to a synthetic magnetite standard. The FTIR-ATR spectra of ferrihydrite bioreduced by *P. delaneyi* also showed possible cell-associated organics such as exopolysaccharides. Such combined detections of biomineral assemblages and organics might serve as biomarkers for hyperthermophilic Fe(III) reduction. With lepidocrocite, *P. delaneyi* produced primarily a ferrous carbonate phase reminiscent of siderite, and with akaganeite, magnetite and a ferrous phosphate phase similar to vivianite were formed. *P. islandicum* showed minor biogenic production of a ferrous phosphate similar to vivianite when grown on lepidocrocite, and a mixed valent phosphate or sulfate mineral when grown on akaganeite. These results expand the range of biogenic mineral transformations at high temperatures and identify spacecraft-relevant spectroscopies suitable for discriminating mineral biogenicity.

## 1. Introduction

Planetary science missions are working to identify the habitability potential and evidence of extinct or extant microbial life on extraterrestrial bodies by searching for organic molecules (Navarro-González *et al.*, 2010; Freissinet *et al.*, 2015; Eigenbrode *et al.*, 2018) and geochemically habitable environments (Grotzinger *et al.*, 2014). However, searching for organic signatures is challenging because many organic molecules degrade over time when exposed to ionizing cosmic rays (Hassler *et al.*, 2014).

An alternative to using organic molecules is to search for signals arising from biogenic mineralogy associated with microbial life. Biogenic minerals can persist for millions to billions of years and aid in the preservation of life-associated organic matter (Keil and Mayer, 2014). Most of what is known about the earliest organisms on Earth comes from unique sedimentary structures made by mats of organisms (Schopf *et al.*, 2007; Noffke *et al.*, 2013) or banded iron formations, shaped at least in part by microbial activity (Cloud, 1973; Widdel *et al.*, 1993; Konhauser *et al.*, 2005; Johnson, 2019).

Characterization of biogenic mineral products has variously been approached using electron microscopy; X-ray diffraction (XRD); and Mössbauer, Raman, and Fourier-transform infrared (FTIR) spectroscopies, but few studies have used these techniques together in ensembles to provide complementary information about particle size and distribution of mineral species (*e.g.,* coatings vs. interiors of grains). The latter three methods are common among the payloads of past and current planetary science missions and will be used in future missions as well. Reflectance spectroscopy is the principal tool used in remote sensing of planetary surfaces.

Raman is being used on several ongoing Mars missions, including the SHERLOC (Scanning Habitable Environments with Raman and Luminescence for Organics and Chemicals) and SuperCam instruments on the NASA Mars 2020 Perseverance rover (Beegle *et al.*, 2015; Wiens *et al.*, 2017) and the Raman Laser Spectrometer on the ESA/Roscosmos ExoMars rover (Rull *et al.*, 2017). Mössbauer spectrometers were used on the Spirit and Opportunity rovers (Klingelhöfer *et al.*, 2003) to understand iron mineralogy of the martian surface (Klingelhöfer *et al.*, 2004; Morris *et al.*, 2004, 2006).

To distinguish mineral biosignatures from abiotic signatures on Earth and elsewhere, a better understanding of microbe–mineral interactions is needed. Among these processes, iron-, sulfur-, and hydrogen-based chemolithoautotrophic metabolisms sustained by mineral interactions make the best candidates to examine life's potential elsewhere (Michalski *et al.*, 2018). Most of what has been previously described for Fe(III) reduction comes from model mesophilic Fe(III)-reducing bacteria such as the genera *Geobacter* and *Shewanella*; these are reported to form magnetite, goethite, hematite, green rust, siderite, chukanovite, and vivianite (Fredrickson *et al.*, 1998, 2003; Ona-Nguema *et al.*, 2002; Zachara *et al.*, 2002; Glasauer *et al.*, 2003; Zegeye *et al.*, 2005, 2007, 2010; O'Loughlin *et al.*, 2007, 2010, 2015, 2019; Salas *et al.*, 2010; Byrne *et al.*, 2011; Piepenbrock *et al.*, 2011; Etique *et al.*, 2016; Markovski *et al.*, 2017; Notini *et al.*, 2019).

Hyperthermophilic Fe(III) reduction, which may have sustained life on the early Earth (Vargas *et al.*, 1998) and conceivably something similar in the martian subsurface, occurs in deep-sea hydrothermal vents and terrestrial hot springs and relies on extracellular reduction of Fe(III) to Fe(II). The terminal electron acceptors for hyperthermophilic iron reduction are commonly nanophase Fe(III) (oxyhydr)oxides, which are ubiquitous in many terrestrial and marine hydrothermal environments on Earth (Kristall *et al.*, 2006; Kozubal *et al.*, 2012; Toner *et al.*, 2012, 2016; Lin *et al.*, 2014, 2016b) and in extraterrestrial environments such as on Mars (Bell *et al.*, 2000; Klingelhöfer *et al.*, 2004; Morris *et al.*, 2004, 2006).

Previously, the hyperthermophilic crenarchaea *Pyrodictium delaneyi* and *Pyrobaculum islandicum* were tested for their ability to grow on a range of Fe(III) (oxyhydr)oxides (Kashyap *et al.*, 2018). Both organisms grew the fastest and produced the most Fe(II) when grown on ferrihydrite. There was also modest growth and Fe(II) production on akaganeite and lepidocrocite and poor growth on goethite, hematite, and maghemite. Both organisms reduced ferrihydrite to magnetite, based on the results of XRD, Mössbauer spectroscopy, and selected-area electron diffraction (Kashefi *et al.*, 2008; Lin *et al.*, 2014; Kashyap *et al.*, 2018), and required direct contact with the mineral for reduction (Feinberg *et al.*, 2008; Kashyap and Holden, 2021).

However, mineral end products for hyperthermophiles grown on akaganeite and lepidocrocite are unknown, and none of the mineral end products have been examined with reflectance or Raman spectroscopies. In this study, hyperthermophilic biogenic mineral transformations of ferrihydrite, akaganeite, and lepidocrocite were characterized using complementary analyses with visible-near infrared (VNIR), Fourier-transform infrared attenuated total reflectance (FTIR-ATR), Raman, and Mössbauer spectroscopies. Mineral end products were compared with abiotic mineral transformations in identical growth media that were heated at the same temperature and duration as the biotic samples and unheated. Identifying specific mineral assemblages associated with bioreduction that are distinguishable from abiotic mineral phases, as detected by complementary spacecraft-relevant spectroscopies, helps to facilitate the search for life in our solar system.

## 2. Materials and Methods

### 2.1. Organisms used

*P. delaneyi* Su06^T^ (DSM 28599) and *P. islandicum* GEO3^T^ (DSM 4184) were used in the present study and obtained from the Deutsche Sammlung von Mikroorganismen und Zellkulturen (DSMZ). *P. delaneyi* is a marine hydrogenotrophic crenarchaeon isolated from a deep-sea hydrothermal vent (Lin *et al.*, 2014, 2016a). *P. islandicum* is a freshwater, facultatively autotrophic crenarchaeon isolated from a solfataric geothermal pool (Huber *et al.*, 1987). In this study, *P. islandicum* was grown heterotrophically.

### 2.2. Mineral synthesis

Nanophase ferrihydrite (two-line), akaganeite, and lepidocrocite were synthesized as previously described (Kashyap *et al.*, 2018; Sklute *et al.*, 2018). Their identity and purity were confirmed by transmission electron microscopy; powder XRD; and VNIR, FTIR-ATR, Raman, and Mössbauer spectroscopies. All synthesized minerals were stored in distilled and deionized water at 4°C to maintain their size and crystallinity properties.

### 2.3. Growth conditions and experimental setup

*P. delaneyi* and *P. islandicum* were each grown with 100 mmol L^−1^ of synthetic nanophase ferrihydrite, akaganeite, and lepidocrocite separately as the terminal electron acceptor as previously described (Supplementary Information) (Selig and Schönheit, 1994; Kashefi *et al.*, 2002; Kashyap *et al.*, 2018). *P. delaneyi* was grown on H_2_ and 0.02% (w/v) yeast extract with 200 kPa of H_2_-CO_2_ (80%:20%) in the headspace. *P. islandicum* was grown on 0.05% (w/v) casein hydrolysate and 0.02% yeast extract with 200 kPa of N_2_ in the headspace. The pH of the media was adjusted to 6.80 ± 0.05 for both *P. delaneyi* and *P. islandicum*.

All incubations were carried out in 160 mL serum bottles containing 50 mL of medium, which sealed with butyl rubber stoppers. All media, including uninoculated controls, were reduced with 0.5 m*M* cysteine-HCl and supplemented with 1.3 m*M* FeCl_2_∙2H_2_O before inoculation or experiment initiation. Each growth medium was inoculated in duplicate with a logarithmic growth-phase culture that had been transferred at least three times on that medium and terminal electron acceptor. *P. delaneyi* and *P. islandicum* were incubated at 90°C and 95°C, respectively. In addition to the biotic samples (heat, cells), there was an uninoculated control (heat, no cells) for each organism and terminal electron acceptor that was incubated at the same temperature and duration as the biotic samples. Finally, another uninoculated control (no heat, no cells) was maintained for the same duration as the biotic sample but not incubated at high temperature.

### 2.4. Sample preparation for spectral analyses

Biogenic and abiotic mineral end products were collected from all samples once the biological samples reached late logarithmic-to-early stationary growth phase. Samples were then prepared for spectral analyses by filtering each sample onto a 0.2 μm pore size polycarbonate filter using a vacuum pump in an anoxic chamber (unless otherwise stated). Each filter was dried inside the anoxic chamber and placed in a gastight container filled with N_2_. Each anoxic container was opened just before spectral acquisition.

For Mössbauer spectroscopy, dried samples were mounted inside the anoxic chamber to minimize O_2_ exposure. Each sample was prepared by mixing ∼50 mg of mineral with 250 mg of table sugar with an agate mortar and pestle. Once a homogeneous powder was obtained, it was mounted into plastic washers backed with Kapton polyimide film tape. Mounts were transported from the anoxic chamber to the Mössbauer facility in a gastight container filled with N_2_. After inserting the sample in the instrument, the chamber was flushed and filled with He gas. Spectral acquisition then began immediately, starting with the coldest temperature measurement and then moving successively warmer.

### 2.5. Spectral data acquisition and analyses

Detailed descriptions of data acquisition parameters and spectral analyses are provided in the Supplementary Information and only briefly summarized here. VNIR spectroscopy was performed with an Analytical Spectral Devices FieldSpec 4 Max spectrometer with incidence and emission angles set to 30° and 0°, respectively. Peak positions were determined after continuum removal and based on local minima and maxima using the OPUS 7.7 software package (Bruker Optics, Inc.).

FTIR-ATR spectroscopy used a Bruker Vertex 70 FTIR equipped with a diamond ATR accessory, an ultra-wide range beam splitter (6000–30 cm^−1^), and a wide-range DLATGS detector. For analysis, continuum removal was performed using a concave rubber band baseline removal algorithm (Wartewig, 2003), and absorption positions were determined using local minima in the OPUS 7.7 software package. For broad bands, peak positions were identified by Gaussian and Lorentzian curve fitting algorithms in MagicPlot v.2.9 (MagicPlot Systems, LLC).

Raman spectroscopy used a Bruker Senterra II Raman microscope with a 532 nm excitation laser and a 20 × objective at Bruker Optics. Spectral data were collected using either 0.25 or 2.5 mW laser power depending on the sensitivity of the sample to phase transformation. All Raman data were baseline-corrected with an asymmetric least squares algorithm (Eilers and Boelens, 2005) and min–max normalized before spectral fitting for feature identification. They were smoothed with the Savitzky–Golay algorithm for visual representation, but no features were assigned based on smoothed data. Band positions were identified using Gaussian and Lorentzian curve fitting algorithms in MagicPlot v.2.9.

Mössbauer spectroscopy used a WEB Research Co. (now SEE Co.) model WT302 spectrometer equipped with a source of ∼40 mCi ^57^Co in Rh and a Janis closed cycle He cooling system (Edina, MN). Spectral data for each sample were acquired over either ±4 mm/s or ±10 mm/s velocity range depending on the extent of magnetic ordering in the sample. Acquisition temperatures were 295K, 220K, 150K, 80K, and 4K. The Mössbauer data were fitted using either Mex_disd or Mex_field (De Grave and Van Alboom, 1991).

## 3. Results

Cells were grown until they reached late logarithmic-to-early stationary growth phase for each mineral. *P. delaneyi* and *P. islandicum* produced 20–25 m*M* Fe^2+^ and 6 m*M* Fe^2+^ above Fe^2+^ concentrations in heated abiotic controls, respectively, when grown on ferrihydrite. Both organisms produced 2–3 m*M* Fe^2+^ above heated abiotic controls when grown on lepidocrocite and akaganeite.

### 3.1. VNIR analysis

In most cases, spectral contrast and overall reflectance drastically decreased for bioreduced minerals relative to abiotic controls as well as starting minerals. These changes were consistent with the formation of visibly darker phases upon bioreduction (Table 1). Detailed analysis of the VNIR spectra is also provided in the Supplementary Information; salient findings are summarized below.

**Table 1.** Key Differences Between Bioreduced and Abiotic Visible–Near-Infrared Spectra for *Pyrodictium delaneyi* and *Pyrobaculum islandicum*

Mineral starting phase	Experimental condition	P. delaneyi				P. islandicum			
		Color and magnetism phase assignment	Peak position changes, μm	0.475–0.550 μm slope	0.86–1.3 μm slope	Color and magnetism Phase assignment	Peak position changes, μm	Peak position changes, μm	0.86–1.3 μm slope
Ferrihydrite	Bioreduced	Black magnetic magnetite	Spectrally flat low reflectance	neg	neut	Black magnetic magnetite	Spectrally flat low reflectance	neut	neut
	Abiotic heated control	Dark reddish-brown, nonmagnetic ferrihydrite with decreased spectral contrast and decreased reflectance reddish-brown nonmagnetic	1.4(ms)	pos	neut	Dark reddish-brown, nonmagnetic ferrihydrite with decreased spectral contrast	1.4(ms)	pos	neg
	Abiotic unheated control	Reddish-brown nonmagnetic ferrihydrite with decreased spectral contrast and decreased reflectance	1.4(ms)	pos	Less pos	Reddish-brown nonmagnetic ferrihydrite	1.43	pos	pos
Lepidocrocite	Bioreduced	Dark orange-brown, nonmagnetic spectrally dark	0.794(ms)	Less pos	neut	Light-brown nonmagnetic spectrally dark	0.794(dc)	Less pos	neut
	Abiotic heated control	Light orange-brown, nonmagnetic lepidocrocite with decreased spectral contrast	0.623(dc), 0.623(dc), 0.978(dc)	pos	Less pos	Light orange-brown, nonmagnetic lepidocrocite	0.593(un), 0.802(un), 0.966(un)	pos	pos
	Abiotic unheated control	Bright yellow-orange, nonmagnetic lepidocrocite	0.593(un), 0.802(un), 0.966(un)	pos	pos	Bright yellow-orange, nonmagnetic lepidocrocite	0.593(un), 0.802(un), 0.966(un)	pos	pos
	Bioreduced	Dark-brown magnetic magnetite or maghemite	0.502(un), 0.728(ms), 0.970(ms)	0.970(ms)	neg	Light-brown nonmagnetic spectrally dark	0.502(un), 0.728(ms), 0.942	Less pos	neut
Akaganeite	Abiotic heated control	Light-brown nonmagnetic akaganeite with decreased spectral contrast	0.502(un), 0.728(dc), 0.942	pos	Less pos	Light-brown nonmagnetic akaganeite with decreased spectral contrast	0.502(un), 0.728(dc), 0.961	pos	Less pos
	Abiotic unheated control	Yellow nonmagnetic akaganeite	0.502(un), 0.728(un), 0.970(un)	pos	pos	Yellow nonmagnetic akaganeite	0.502(un), 0.728(un), 0.970(un)	pos	pos

dc = decreased; ms = missing; neg = negative; neut = neutral; pos = positive; un = unchanged.

**FIG. 1.**
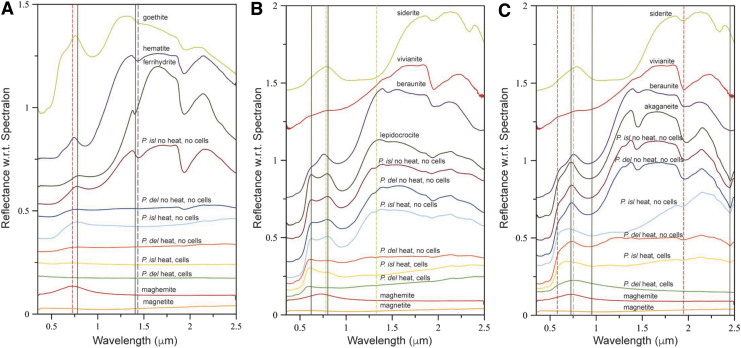
VNIR spectra of biotic (heat, cells), heated abiotic (heat, no cells), and unheated abiotic (no heat, no cells) mineral transformations of ferrihydrite **(A)**, lepidocrocite **(B)**, and akaganeite **(C)** for *Pyrodictium delaneyi* (*P. del*) and *Pyrobaculum islandicum* (*P. isl*) along with iron mineral reference standards. All spectra are offset for clarity. VNIR = visible–near-infrared.

Ferrihydrite that was bioreduced by *P. delaneyi* and *P. islandicum* was spectrally flat and minimally reflective, indicating the presence of a highly absorbing and opaque phase like magnetite (Fig. 1A and Table 1). Spectra for lepidocrocite that was bioreduced by *P. delaneyi* and *P. islandicum* were visibly darkened, losing many of the distinctive absorptions typical of lepidocrocite (Fig. 1B). The maximum in the visible region at 0.794 μm for lepidocrocite was completely lost upon bioreduction by *P. delaneyi.* In contrast, the position for this feature in *P. islandicum* was unchanged, although reflectance at this wavelength was drastically reduced. Spectra for akaganeite that were bioreduced by *P. delaneyi* appeared significantly muted, unlike any of the abiotic or reference spectra (Fig. 1C). The resultant phase was similar to magnetite or maghemite. Bioreduced akaganeite for *P. islandicum* also showed a decrease in spectral contrast and overall reflectivity relative to the akaganeite reference and the unheated abiotic control. It retained the relative positions of the 2(^6^A1 → ^4^T1) transition at ∼0.5 μm but lost the distinctive maximum in the visible region at ∼0.728 μm for akaganeite.

Spectral slopes in the 0.475–0.550 and 0.86–1.3 μm (Supplementary Fig. S1 and Table 1) ranges are often associated with Fe^3+^ and Fe^2+^ electronic transitions and are distinctive based on mineral and redox state (Dyar *et al.*, 2018, 2019). Here, slopes among sample spectra were useful in distinguishing not only redox states but also the extents of transformation, highlighting subtle differences for the two organisms. Positive slopes typically corresponded with Fe^3+^ phases, while negative or neutral slopes with Fe^2+^ or mixed Fe^3+^−Fe^2+^ phases. Detailed discussion of these differences is provided in the Supplementary Information.

### 3.2. FTIR-ATR analysis

FTIR-ATR data highlight key differences in iron mineralogy and display potential cell-associated features that are distinctive between bioreduced and abiotic conditions (Table 2). FTIR-ATR spectral analyses were based on the maxima of Gaussian or Lorentzian peak fitting results for these spectra rather than the maxima as they appear in the spectra (Table 2). Detailed analysis of the FTIR-ATR spectra is provided in the Supplementary Information.

**Table 2.** Select Peak Positions (cm^−^^1^) for Bioreduced and Abiotic Fourier-Transform Infrared Attenuated Total Reflectance Spectra for *Pyrodictium delaneyi* and *Pyrobaculum islandicum*

Mineral starting phase	Experimental condition	P. delaneyi		P. islandicum	
		Phase assignment	Peak positions, cm^−1^	Phase assignment	Peak positions, cm^−1^
Ferrihydrite	Bioreduced	Magnetite (cation deficient)	**320**(sh), **542**(sh)	Magnetite	**566**(dp), **320**(ms)
		Cell-associated or mineral phosphates	**954, 1035, 1093**(br), ∼450(ms)	Cell-associated or mineral phosphates	**926, 1022, and 1101**(br)
		Possibly EPS	(**1239, 1342, 1520, 1619**) (br)		
	Abiotic heated control	Ferrihydrite altered	583(dp), 631(ms), 703	Ferrihydrite altered	**573**(dp), 631(ms), 693
		Medium-associated or mineral	(**970, 1048, 1107**) (br)	Medium-associated or mineral-sorbed phosphate	(**943, 1025, 1101**) (br)
	Abiotic unheated control	Ferrihydrite altered	596(dp), 682, 631	Ferrihydrite altered	594(dp), 685, 631
		Medium-associated or mineral sorbed phosphate	(**965, 1042, and 1099**) (br)	Medium-associated or mineral-sorbed phosphate	(**972, 1054, 1105**) (br)
Lepidocrocite	Bioreduced	Siderite	**730, 860**(sh), **1400**(br)	Vivianite or beraunite	**528**(dp), (**1016, 1021, 1157**) (br), 346, 263, 216
	Abiotic heated control	Lepidocrocite altered	470, 612, 635(dp), 754, 1133	Lepidocrocite altered	474, 514(dp)
		Phosphate mineral or medium-associated	∼500(ms), ∼780(ms), **1021**(sh), **1022**(br), **1133**(br)	Medium-associated or mineral-sorbed phosphate	**1013**(br), **1020**(sh), **1154**(br)
	Abiotic unheated control	Lepidocrocite altered	468, 611, 635(dp), 755, 1146	Lepidocrocite altered	476, 514(dp)
		Medium-associated or mineral sorbed phosphate	∼500(ms), ∼780(ms), **1021**(br), **1022**(sh), **1146**(br)	Medium-associated or mineral-sorbed phosphate	**1020**(sh), **1023**(br), **1149**(br)
Akaganeite	Bioreduced	Magnetite	**566**	Phosphate mineral or cell associated and growth medium-sorbed phosphate	575(dp), **1016**(br)**, 1037, 1189**
		Vivianite or cell-associated phosphate sorbed on mineral or phosphorus in magnetite akaganeite altered	**944, 980, 1050**	Akaganeite altered	∼650(dc)
		Akaganeite altered	∼650(dc)		
	Abiotic heated control	Medium-associated or mineral sorbed phosphate	**1066**(br)	Medium-associated or mineral-sorbed phosphate	**981, 1030, 1189**
		Akaganeite altered	∼650(dc)	Akaganeite altered	∼650(dc)
	Abiotic unheated control	Akaganeite altered	**360**(dp), 817	Akaganeite altered	**356**(dp), ∼650(dc), 814

Bold values are features associated with new phases.

br = broad; dp = deep(er); EPS = extracellular polymeric substances; sh = sharp.

**FIG. 2.**
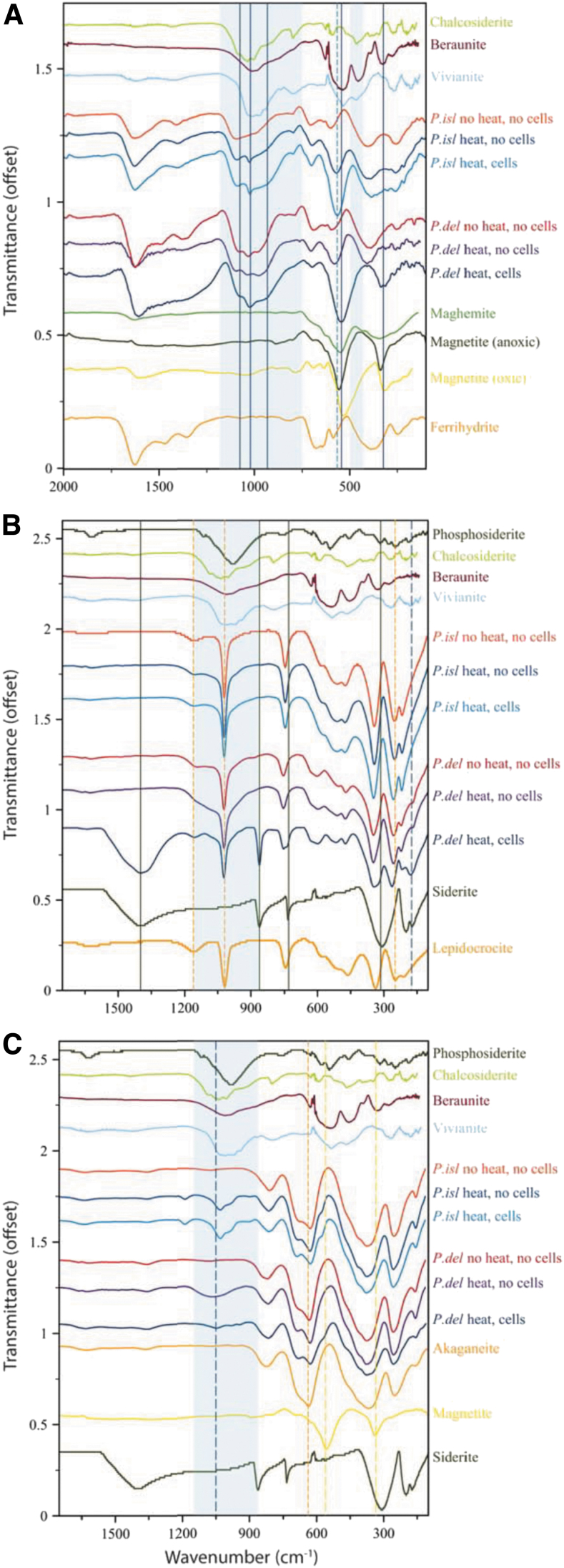
FTIR-ATR spectra of biotic (heat, cells), heated abiotic (heat, no cells), and unheated abiotic (no heat, no cells) mineral transformations of ferrihydrite **(A)**, lepidocrocite **(B)**, and akaganeite **(C)** for *Pyrodictium delaneyi* (*P. del*) and *Pyrobaculum islandicum* (*P. isl*) along with iron mineral reference standards. All spectra are continuum removed and offset for clarity. Blue shaded box represents regions of phosphate absorptions, whereas solid/dashed lines represent key features and are color coded in the same manner as sample/reference spectra. FTIR-ATR = Fourier-transform infrared attenuated total reflectance.

Upon bioreduction of ferrihydrite, both organisms formed a black magnetic product that was spectrally consistent with magnetite (Fig. 2A). The spectrum of ferrihydrite bioreduced by *P. delaneyi* had sharp absorption positions at 542 and 320 cm^−1^, and band envelopes with shapes most similar to an aerobically filtered magnetite (magnetite [oxic]) or a cation-deficient magnetite. The bioreduced ferrihydrite spectrum for *P. islandicum* shows a distinctly deeper absorption at 566 cm^−1^, lower than what is typical for ferrihydrite at 583 cm^−1^, but higher than that for synthetic nanophase magnetite and maghemite. Moreover, unlike *P. delaneyi*, *P. islandicum* lacked a sharp and isolated absorption feature at ∼320 cm^−1^.

Rather, it retained the position of ferrihydrite absorptions at 387 and 251 cm^−1^, although the overall spectral shape of this region changed compared with unheated and heated abiotic controls. This suggests potential contributions due to another phase not present in the controls. Based on the reference spectra, this phase could be magnetite, maghemite, or an iron phosphate because they all have features in this region. Bioreduced, unheated, and heated-control spectra for both *P. delaneyi* and *P. islandicum* grown on ferrihydrite show broad absorption bands in the 900–1200 cm^−1^ region, indicative of symmetric and antisymmetric stretching vibrations of phosphates and hydrogen phosphates (Fig. 2A) (Frost *et al.*, 2002, 2014). These bands varied in shape and position among conditions and organisms.

The 1200–1700 cm^−1^ region showed the most distinctive features and shifts in the bioreduced ferrihydrite spectrum for *P. delaneyi* (Fig. 2A). Ferrihydrite exhibits three absorption features in this range at ∼1360, ∼1465, and ∼1630 cm^−1^. The first two features represent symmetric and antisymmetric modes of (atmospheric) absorbed carbonate, respectively, and the third represents the deformation mode of H_2_O (Hausner *et al.*, 2009). These three distinct bands became one broad expanded feature in the bioreduced spectrum, suggesting significant contributions other than ferrihydrite to this region.

This band envelope was likely a combination of several abiotic and biotic components, including, but not limited to, (1) adsorbed carbonate on ferrihydrite from a CO_2_ headspace and bicarbonate-buffered medium, (2) HOH-bending modes for phosphate minerals that form, and (3) cell-associated proteins, which typically show features at 1400, 1550, and 1640 cm^−1^ when associated with extracellular polymeric substances (EPS) (Maddela *et al.*, 2018). EPS typically also displays bands for polysaccharides at ∼920, 1020, and 1100 cm^−1^, consistent with the positions noted in the *P. delaneyi* bioreduced ferrihydrite spectrum. For *P. islandicum*, the differences between the abiotic and biotic spectra in this region are subtle and therefore indistinguishable.

Lepidocrocite that was bioreduced by *P. delaneyi* presented convincing evidence of siderite in the FTIR-ATR data (Fig. 2B). Its spectrum exhibited all the distinctive absorptions for this phase at ∼730, 860, and 1400 cm^−1^ due to the in-plane bending mode (ν4), out-of-plane bending mode (ν2), and asymmetric internal stretching mode (ν3) of the carbonate ion, respectively (Dubrawski *et al.*, 1989). Lepidocrocite transformations for *P. islandicum* presented minor differences between bioreduced and the two abiotic control spectra (Fig. 2B and Table 2). All three spectra showed increased peak depth at ∼510 cm^−1^.

Both the heated abiotic control and bioreduced lepidocrocite spectra showed subtle broadening around ∼850–1260 cm^−1^, which was greater in the bioreduced spectrum compared with the heated abiotic control. Moreover, only the bioreduced spectrum showed minor shifts in the lepidocrocite features at ∼340, ∼250, and ∼210 cm^−1^ toward higher wave numbers. Together, these changes were consistent with the formation of a minor phosphate phase similar to vivianite or beraunite [Fe^2+^Fe^3+^ (PO_4_)4(OH)5∙6H_2_O], but only in the bioreduced spectrum. The slightly broadened feature associated with phosphate vibrations in the heated abiotic control was likely due to phosphate moieties in solution or sorbed on lepidocrocite.

Akaganeite that was bioreduced by *P. islandicum* and *P. delaneyi* and the heated abiotic control for *P. islandicum* showed distinct phosphate absorptions in ∼920–1200 cm^−1^ region (Fig. 2C). The heated abiotic control for *P. delaneyi* showed a broad diffuse band in this region, unlike the bioreduced spectrum, which showed three distinct absorptions at 944, 980, and 1050 cm^−1^. In contrast, both the heated abiotic control and bioreduced spectra for *P. islandicum* showed four distinct absorptions at 903, 988, 1032, and 1193 cm^−1^. Spectral subtraction of these two spectra suggested that these phosphate absorptions were deeper in the bioreduced compared with the heated abiotic control spectrum.

However, this could also be due to subtle differences in contact of the sample with the ATR crystal, which can change depth of features. While the phosphate absorptions were reminiscent of vivianite, they were not a perfect match. It is also possible that these features represented biological phosphate moieties binding on a mineral surface (Parikh and Chorover, 2006; Cagnasso *et al.*, 2010; Parikh *et al.*, 2014) or structural phosphorus in magnetite (Jurado *et al.*, 2003). Bioreduced akaganeite in *P. delaneyi* showed a small but distinct shoulder at ∼566 cm^−1^, consistent with magnetite. An absorption at 575 cm^−1^, ∼10 cm^−1^ greater than that noted for *P. delaneyi*, was also present in the *P. islandicum* heated abiotic control and bioreduced spectra. However, given its shift to higher wave numbers compared with magnetite, this most likely represented out-of-plane bending in phosphates (Frost *et al.*, 2002).

### 3.3. Raman analysis

**FIG. 3.**
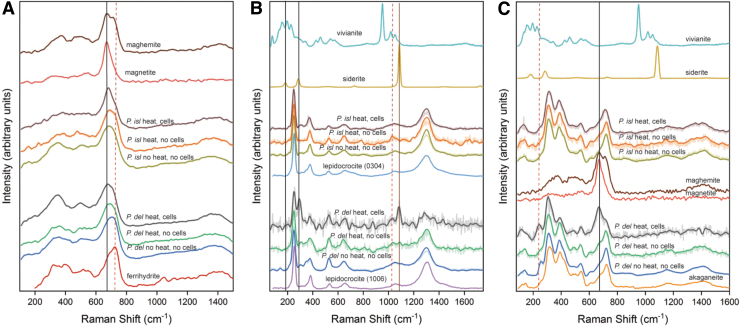
Raman spectra of biotic (heat, cells), heated abiotic (heat, no cells), and unheated abiotic (no heat, no cells) mineral transformations of ferrihydrite **(A)**, lepidocrocite **(B)**, and akaganeite **(C)** for *Pyrodictium delaneyi* (*P. del*) and *Pyrobaculum islandicum* (*P. isl*) along with iron mineral reference standards. Spectra are baseline corrected and min–max normalized. Smoothed spectra are overlaid on raw spectra for clarity. Two different synthetic lepidocrocite minerals, (1006) and (0304), were used for growth of *P. delaneyi and P. islandicum*, respectively, and both are shown as reference **(B)**.

Raman spectra for each sample (Fig. 3) and fitted band positions (Table 3) showed that *P. delaneyi* had more distinctive mineral transformations upon microbial reduction than *P. islandicum* and that different mineral phases formed depending on the electron acceptor. Raman spectral analyses are based on peak fitting results for these spectra rather than the maxima as they appear in the spectra (Table 3). Additional analysis of the Raman spectra is provided in the Supplementary Information.

**Table 3.** Raman Band Positions Based on Curve Fitting Analysis of Reference Standards, Bioreduced, and Abiotic Control Spectra of *Pyrodictium delaneyi* and *Pyrobaculum islandicum* Grown on Three Different Iron Oxides

Mineral starting phase	Organism	Mineral reference standard/experimental condition	Raman band position, cm^−1^
		Ferrihydrite (f)	**327, 409**, **528,** 631, **695**, **735**
		Maghemite (mh)	318, **374**, **496**, 646, **670, 720**
		Magnetite (mt)	**330**, 450, **539**, **668**, 702
		Akaganeite (a)	253, **318**, **389**, 428, 495, **545**, 613, 679, **725**, 878, 1165, 1394
		Lepidocrocite (l)	**254**, 321, 357, **382**, **532**, **654**, 1057, **1302**
		Vivianite (v)	79, **155**, **197**, **229**, 287, 343, 425, 462, 543, 589, 828, **952**, **1016**, **1055**
		Siderite (s)	**182**, **285**, 514, 730, **1086**, 1439
Ferrihydrite	*P. delaneyi*	Abiotic unheated control	156, **351**, **515**, 589, **690**(f), **730**(f)
		Abiotic heated control	167, **347**, **507**, 585, **685**(f), **731**(f)
		Bioreduced	205, **344**, **496**, 584, **677**(mt), 721(mt)
	*P. islandicum*	Abiotic unheated control	155, **343**, **508**, 576, **683**(f), 728(f)
		Abiotic heated control	224, **362**, 476, **532**, **688**(f), **733**(f)
		Bioreduced	226, **385**, **536**(mt), 624, **682**(mt), 731(f)
Akaganeite	*P. delaneyi*	Abiotic unheated control	253, **313**(a), **385**(a), 422, 491, **540**(a), 610, 679, **721**(a), 873, 1040(gp), 1156, 1394
		Abiotic heated control	248, **311**(a), **387**(a), 423, 493, **538**(a), 607, 673, **720**(a), 869, 1062(gp), 1161, 1394
		Bioreduced	**241**, **311**(mt), **388**(a), 485, 541, **668**(mt), 721, 1017(cp), 1358
	*P. islandicum*	Abiotic unheated control	253, **313**(a), **383**(a), 421, 490, **541**(a), 615, 682, **721**(a), 860, 1025(gp), 1158, 1401
		Abiotic heated control	250, **310**(a), **388**(a), 426, 490, **540**(a), 610, 675, **723**(a), 859, 1030(gp), 1162, 1399
		Bioreduced	248, **313**(a), **384**(a), 418, 482, **540**, **673**(mh/a), **709**(mh), **730**(a), 1068(cp), 1162, 1381
Lepidocrocite	*P. delaneyi*	Abiotic unheated control	**250**(l), 307, 346, **379**(l), **529**(l), **644**(l), 1058, **1295**
		Abiotic heated control	**250**(l), 308, 341, **379**(l), **529**(l), **644**(l), 799, 1073, **1300**(l)
		Bioreduced	**252**(l), **294**(s), 339, 377, 528, 647, 824, 1026, **1084**(s), **1303**(l)
	*P. islandicum*	Abiotic unheated control	**250**(l), 312, 348, **377**(l), **528**(l), **645**(l), 803, 1053, **1298**(l)
		Abiotic heated control	**250**(l), **376**(l), **528**(l), **648**(l), 776, 1042, **1297**
		Bioreduced	**249**, 309(p), 341, **373**(l), 527, **645**(l), 796, 1036(p), **1297**(l)

Positions highlighted in bold are the most intense bands in each fitted spectrum.

cp = cell-associated phosphate; gp = growth medium associated phosphate; p = Fe^2+^ or mixed Fe^2+^−Fe^3+^ phosphate mineral.

Ferrihydrite that was bioreduced by *P. delaneyi* showed the lowest wave number band positions (677 and 721 cm^−1^) in the 650–730 cm^−1^ region, indicating a positional shift toward magnetite. *P. delaneyi* unheated and heated abiotic control spectra showed both shapes and positions consistent with ferrihydrite, although with a slight shift toward lower wave numbers when compared with the reference standard (Fig. 3A). These features can be explained as a combination of ferrihydrite and magnetite, an intermediate phase, or possibly a cation-deficient magnetite.

Ferrihydrite bioreduced by *P. islandicum* showed a very subtle positional shift toward lower wave numbers, but a distinctively sharp Lorentzian peak shape for its main feature at 682 cm^−1^, indicative of magnetite, while *P. islandicum* unheated and heated abiotic control spectra were consistent with ferrihydrite in shape and position (Fig. 3A).

Lepidocrocite that was bioreduced by *P. delaneyi* showed two prominent and unique features relative to the abiotic controls and reference lepidocrocite at 294 and 1084 cm^−1^ (Fig. 3B and Table 3). These features were consistent with the lattice and symmetric stretching modes of a ferrous carbonate phase, although the intensity ratio for these two features is unexpected for a siderite (Table 3) (Hanesch, 2009; Sharma *et al.*, 2009). In addition to siderite, it is possible that the band at 294 cm^−1^ could be associated with a FeO stretching mode unique to bioreduction process. Lepidocrocite bioreduced by *P. islandicum* showed only subtle changes relative to the starting mineral (Fig. 3B), namely a gradual shift in expected features of lepidocrocite at 382 and 1057 cm^−1^ to lower wave numbers. It is possible that a ferrous or mixed ferric–ferrous phosphate phase contributed to the shifted position.

Akaganeite that was bioreduced by *P. delaneyi* showed three notable distinctions from the abiotic controls and reference akaganeite: a sharp feature at 668 cm^−1^ consistent with magnetite, a band at 241 cm^−1^, and a marked increase in the relative band intensity of 311:387 cm^−1^ features typically associated with FeO stretching vibrations (Fig. 3C). This increase in relative intensity was due to the overlapped presence of a feature at 310 cm^−1^ associated with the T2g mode of magnetite. In contrast, akaganeite bioreduced by *P. islandicum* showed two distinctive features when compared with the abiotic controls and reference akaganeite: a broader peak at ∼700 cm^−1^ and a fitted band at 1068 cm^−1^ under the broad feature at ∼1160 cm^−1^ (Fig. 3C and Table 3).

Curve fitting suggested that the broader band at ∼700 cm^−1^ was likely due to a combination of maghemite in addition to akaganeite in this region. The feature at 1068 cm^−1^ can be expected for a stretching vibrational mode of phosphate, either mineral or cell-associated, but lacked typical band positions based on reference phosphate standards (Table 3 and Supplementary Fig. S2).

### 3.4. Mössbauer analysis

**FIG. 4.**
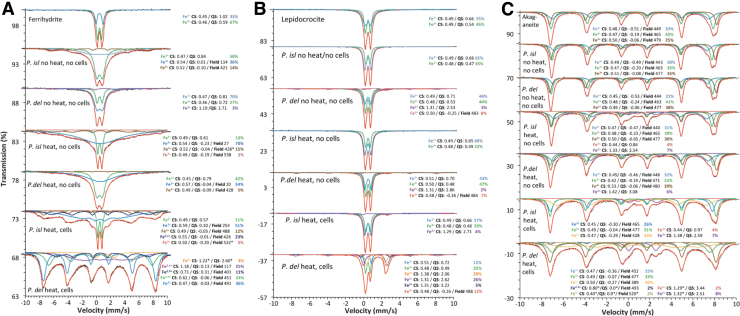
Mössbauer spectra at 80K of biotic (heat, cells), heated abiotic (heat, no cells), and unheated abiotic (no heat, no cells) mineral transformations of ferrihydrite **(A)**, lepidocrocite **(B)**, and akaganeite **(C)** for *Pyrodictium delaneyi* (*P. del*) and *Pyrobaculum islandicum* (*P. isl*) along with starting mineral reference standards. Each spectrum is shown along with its associated fits and parameters, which are color coded, scaled, and offset for clarity. Mössbauer parameters for CS and QS are in mm/s with respect to α-Fe foil at 295K, and hyperfine field (F) is the highest probability value of the hyperfine field distribution and is given in kOe.

For *P. delaneyi*, the bioreduced ferrihydrite displayed significant Fe^2+^ in Mössbauer spectra, which could be fit at 295K with two Fe^2+^ and one broad Fe^2.5+^ distributions (Fig. 4A and Supplementary Fig. S3A). At all other temperatures, overlap with magnetically ordering phases made only one Fe^2+^ distribution resolvable (4% spectral area; Supplementary Fig. S3). Magnetic ordering of Fe^2.5+^ and a portion of the Fe^3+^ below 220K are consistent with nanophase magnetite (Supplementary Fig. S3B). While the distributions assigned to magnetite did not show typical parameters for that phase above the Verwey transition, by 4K, the spectrum closely resembled an admixture of ferrihydrite and nanophase magnetite (Supplementary Fig. S3D).

Additionally, a completely unrelated and unidentifiable Fe^2+^ phase was present in this sample, as evidenced by the persistence of a Fe^2+^ doublet even at low temperatures (Fig. 4A and Supplementary Fig. S3). Previous Mössbauer characterization of ferrihydrite reduced by *P. delaneyi* showed a similar small Fe^2+^ contribution (CS = 1.26 mm/s, QS = 2.80 mm/s), which was attributed to a Fe^2+^ phosphate mineral, similar to vivianite (Lin *et al.*, 2014). For *P. islandicum*, a Fe^2.x+^ (0.9 > x > 0.5) feature could be resolved in the 295K spectrum (Supplementary Fig. S3A). This distribution gained more Fe^3+^ character as it magnetically ordered with decreasing temperature, likely due to admixing of other strictly Fe^3+^ components (*i.e.,* ferrihydrite), which were also magnetically ordering.

This phase, which only formed upon bioreduction, is consistent with a mixture of magnetite and maghemite or with a cation-deficient maghemite of multiple nanoparticulate grain sizes. This phase was not resolvable at 4K due to overlap but clearly changed the shape of the spectral envelope (Supplementary Fig. S3D).

For *P. delaneyi*, the Mössbauer spectra of bioreduced lepidocrocite when compared with abiotic controls show significant transformation. At 295K, ferrous distributions comprise 56% of the spectral area (Supplementary Fig. S4A). The largest contribution was a doublet with CS = 1.24 mm/s and QS = 1.83 mm/s, which is consistent with siderite (Dyar *et al.*, 2006). For *P. islandicum*, the bioreduced Mössbauer spectrum displayed a small Fe^2+^ doublet (3–4% spectral area) in all temperatures down to 80K, which appeared to magnetically order at 4K (Fig. 4B and Supplementary Fig. S4). Due to significant overlap with lepidocrocite doublets, particularly for the left-hand peak of this Fe^2+^ phase, the fitted parameters reported were not unique, and the exact composition of this Fe^2+^ component could not be determined (Fig. 4B and Supplementary Fig. S4).

For *P. delaneyi*, the bioreduced akaganeite spectrum shows clear evidence of magnetite (8–10% spectral area), which was distinctly resolvable from 295K to 150K, but highly overlapped with akaganeite at temperatures below magnetite's Verwey transition (Fig. 4C and Supplementary Fig. S5). Two additional Fe^2+^ components were also present in this spectrum (Fig. 4C and Supplementary Fig. S5A–C). However, due to the highly overlapped nature of these two distributions, the fitted parameters reported were not unique to any specific mineral species. Also, unlike the Fe^2+^ components in the heated abiotic control, not all the Fe^2+^ phases were magnetically ordered at 4K (Supplementary Fig. S5D).

For *P. islandicum*, the bioreduced akaganeite spectrum contains similar distributions to the heated abiotic control with minor additional ferric and ferrous components (Fig. 4C and Supplementary Fig. S5). Another distinctive difference in this spectrum was that the innermost akaganeite Fe^3+^ sextet magnetically ordered much more slowly than in the unheated abiotic control or reference spectra. While little can be concluded based on this difference alone, it was an observable change in this bioreduced spectrum.

## 4. Discussion

**FIG. 5.**
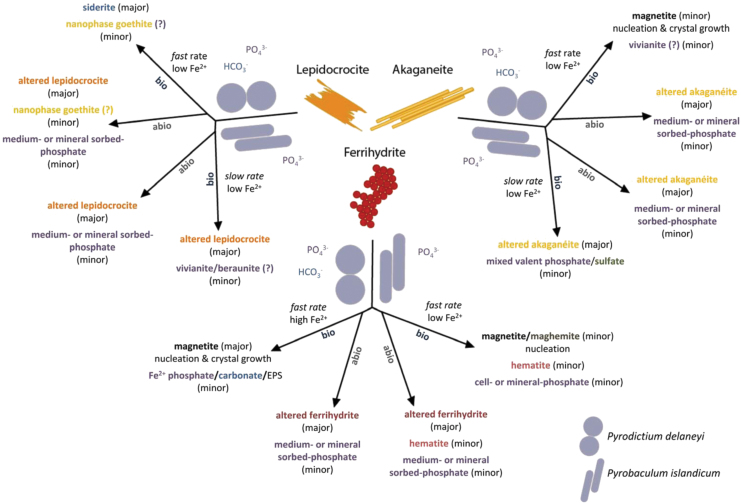
Conceptual model and summary of biotic (bio) and abiotic (abio) mineral transformations of ferrihydrite, lepidocrocite, and akaganeite that occurred in marine (*Pyrodictium delaneyi*) and freshwater (*Pyrobaculum islandicum*) growth media at 90°C and 95°C, respectively.

This study demonstrated that ferrihydrite, lepidocrocite, and akaganeite were biologically reduced by two hyperthermophilic crenarchaea, *P. delaneyi* and *P. islandicum*; that the mineral products varied with the starting mineral, organism, and environmental conditions; and that the products were distinguishable from abiotic mineral phases incubated under similar conditions (Fig. 5). The bioreduction products represent transformations by actively respiring organisms as both *P. delaneyi* and *P. islandicum* were adapted to each Fe(III) (oxyhydr)oxide through more than three serial transfers. It was shown previously that both *P. delaneyi* and *P. islandicum* adapt their physiology to growth on ferrihydrite relative to growth on a soluble terminal electron acceptor and require direct mineral contact for reduction (Feinberg *et al.*, 2008; Kashyap and Holden, 2021).

In addition to physiological adaptation to each mineral, significantly lower initial cell concentrations (10^5^ cells mL^−1^) and shorter timelines for mineral reduction and collection (1–4 days) were used in this study. This ensured that the mineral products examined were the result of active growth from cells adapted to iron that grew and doubled on each mineral rather than due to dying or dead cells serving as organic templates for fortuitous reduction. The bioreduced mineral products included primarily magnetite, a ferrous carbonate similar to siderite, and a ferrous phosphate similar to vivianite. *P. delaneyi* exhibited more extensive and distinctive bioreduced mineral products than *P. islandicum*. The recorded changes in mineralogy were best interpreted when using a complementary combination of VNIR, FTIR-ATR, Raman, and Mössbauer spectroscopies. While each technique alone pointed to possible end products, when taken together, they provided substantial confidence in identified phases.

This study builds on our previous work (Kashyap *et al.*, 2018) in which the rates and extent of ferrihydrite, lepidocrocite, and akaganeite reduction by *P. delaneyi* and *P. islandicum* were determined but not the final mineral end products, nor the associated spectral biosignatures. Final mineral end products were determined previously for hyperthermophiles grown on ferrihydrite (Kashefi *et al.*, 2008; Lin *et al.*, 2014; Amenabar and Boyd, 2018; Kashyap *et al.*, 2018) but not for hyperthermophiles grown on lepidocrocite or akaganeite.

In our previous study, *P. delaneyi* and *P. islandicum* grew to the highest cell concentrations at the fastest growth and Fe^2+^ production rates and produced the most Fe^2+^ when grown on ferrihydrite, although *P. delaneyi* produced significantly more Fe^2+^ (25 m*M*) than *P. islandicum* (6 m*M*). That study showed that both organisms produced 2–3 m*M* Fe^2+^ when grown on lepidocrocite and akaganeite (Kashyap *et al.*, 2018). When related back to the study herein, differences in cell-specific Fe^2+^ production rates and Fe(III) reduction rates were the most predictive for the bioreduction mineral products identified (Fig. 5). Cell-specific Fe^2+^ production rates >20 fmol Fe^2+^ cell^−1^ h^−1^ or Fe(III) reduction rates >58 μ*M* h^−1^ resulted in the most extensive and explicitly detectable mineral transformations (“fast rate” in Fig. 5). In contrast, cell-specific Fe^2+^ production rates and Fe(III) reduction rates <5 fmol Fe^2+^ cell^−1^ h^−1^ and 18 μ*M* h^−1^, respectively, resulted in limited mineral transformations in this study (“slow rate” in Fig. 5).

Differences in size and morphology of phase transformations as well as the amount of Fe^2+^ present in the dissolved versus solid phase during ferrihydrite, lepidocrocite, and akaganeite reduction were also previously reported for *P. delaneyi* and *P. islandicum* (Kashyap *et al.*, 2018). With *P. delaneyi*, spherical mineral grain size increased during bioreduction of ferrihydrite (25–40 nm diameter), lepidocrocite (50–300 nm diameter), and akaganeite (10–250 nm) relative to minerals formed in the abiotic controls. In contrast, *P. islandicum* did not show any larger spherical mineral grain sized end products in the bioreduction end products of ferrihydrite, lepidocrocite, or akaganeite. For both organisms, ferrihydrite reduction yielded a much larger amount of Fe^2+^ in the solid versus dissolved phase, with only ∼3% and ∼7.5% of total Fe^2+^ present in the dissolved phase for *P. islandicum* and *P. delaneyi*, respectively.

Akaganeite and lepidocrocite reductions yielded a larger proportion of dissolved Fe^2+^ in the total Fe^2+^ produced for both organisms. For akaganeite, ∼52% and ∼44% of the total Fe^2+^ was in the dissolved phase for *P. islandicum* and *P. delaneyi*, respectively; when *P. islandicum* and *P. delaneyi* were grown with lepidocrocite, ∼25% and ∼75% of the total Fe^2+^ was in the dissolved phase, respectively (Kashyap *et al.*, 2018). These differences in size, morphology, and proportion of dissolved Fe^2+^ can be used to inform mechanisms of formation of the varied mineral phases that were identified.

A collective examination of the rates and extents of reduction, aqueous Fe^2+^ accumulation, sizing, and spectral characteristics of bioreduction products suggests that mineral transformations were influenced by the amount, flux, and fate of Fe^2+^ produced, which varied with the type and bioavailability of the starting Fe(III) (oxyhydr)oxide (Fig. 5). Both solid-state/topotactic conversion and dissolution–reprecipitation processes were likely involved in secondary mineralization, but which of these two processes dominated depended on Fe^2+^ flux. At a high Fe^2+^ flux and high Fe^2+^ accumulation with minimal dissolved Fe^2+^ as seen in the case of ferrihydrite, solid phase transformation to magnetite occurred.

A greater extent of reduction of ferrihydrite and Fe^2+^ production by *P. delaneyi* relative to *P. islandicum* resulted in both magnetite nucleation and crystal growth, and this was characterized by increased spherical grain sizes among the transformed products. The additional minor amounts of ferrous phosphate and carbonate that also formed when ferrihydrite was bioreduced by *P. delaneyi* likely resulted from dissolution–reprecipitation associated with aqueous Fe^2+^ generated from bioreduction reacting with HCO_3_^−^ and PO_4_^3−^ in the growth medium, or in the case of phosphate, possible reactions with cell associated phosphate moieties. *P. islandicum* grown on ferrihydrite, however, showed a slower rate of reduction with lower Fe^2+^ accumulation, which favored nucleation of magnetite as a minor phase with limited crystal growth.

Conversely, at low Fe^2+^ flux and lower Fe^2+^ accumulation with larger fractions of total Fe^2+^ as dissolved Fe^2+^, secondary mineralization products such as siderite and vivianite formed (Fig. 5). Magnetite also formed, but likely through a different mechanism. For *P. delaneyi*, transformations only occurred when rates of reduction were fast even if Fe^2+^ accumulation was low. Interestingly, magnetite formed from bioreduction of akaganeite by *P. delaneyi* likely occurred via dissolution–reprecipitation and not solid-state conversion, as evidenced by elevated dissolved Fe^2+^ and a drastic increase in crystal size and change in morphology (laths to spheres) (Kashyap *et al.*, 2018). With *P. islandicum* grown on lepidocrocite and akaganeite, slow rates of reduction and low Fe^2+^ accumulation resulted in the formation of minor amounts of ferrous or mineral-sorbed phosphates upon bioreduction. Ferrous carbonate and ferrous phosphate phases such as siderite and vivianite have not been reported previously as iron bioreduction products at high temperatures.

Understanding biosignatures requires analysis of abiotic transformations under the reaction conditions. Abiotic reactions will depend on starting mineral, salt, pH buffer, and other growth medium components (Supplementary Information). Abiotic reactants include small amounts of Fe^2+^ (1.3 m*M* FeCl_2_) added to initiate reduction, cysteine added as reducing agent, and HCO_3_^−^ and PO_4_^3−^ in the growth media. Minor aqueous Fe^2+^ sorbed on poorly crystalline Fe(III) (oxyhydr)oxide surfaces can catalyze transformation to more crystalline phases such as goethite and hematite through reductive dissolution (Tronc *et al.*, 1992; Zachara *et al.*, 2002; Cornell and Schwertmann, 2003; Hansel *et al.*, 2003, 2005). Minor hematite and goethite were observed under select abiotic conditions and thus attributed to abiotic reactions even in bioreduction experiments.

Strong adsorption of phosphate and organics to mineral surfaces is known to prevent crystallization or nucleation of magnetite (Fredrickson *et al.*, 1998; Zachara *et al.*, 2002; Glasauer *et al.*, 2003; O'Loughlin *et al.*, 2010, 2015, 2019). Competitive sorption of Fe^2+^, organics, and phosphate occurred in *P. islandicum* media, resulting in surface passivation of bioavailable sites for reduction, and less extensive secondary mineralization overall compared with *P. delaneyi.* Previous studies have observed siderite as a product of ferrihydrite, akaganeite, and lepidocrocite bioreduction in systems with bicarbonate concentrations more than 20 m*M* (Fredrickson *et al.*, 1998; Roh *et al.*, 2003).

*P. delaneyi* was grown hydrogenotrophically using *∼*30 m*M* bicarbonate. However, siderite only formed in the reduction of lepidocrocite but not akaganeite or ferrihydrite. It is presently unclear why we observe this difference, but it is possible that PO_4_^3−^ sorbs more effectively on lepidocrocite than akaganeite, inhibiting magnetite formation during lepidocrocite reduction. Sorption of PO_4_^3−^ in this manner would promote formation of siderite as typically phosphate ions inhibit carbonate mineral formation (Sánchez-Román *et al.*, 2014).

Reduction due to abiotically generated Fe^2+^ and/or Fe^2+^ phases was additionally geochemically modeled (Supplementary Information). Aqueous solution chemistry for each growth medium predicted saturation of mineral phases that were either not observed or observed but part of different mineral assemblages than those experimentally determined due to bioreduction. Therefore, it can be concluded that the organisms and their metabolic activity directly influenced mineral precipitation/nucleation by creating suitable microenvironments either at the cell surface or in association with EPS.

Selective incorporation of organics and cations into magnetite formed from dissimilatory iron reduction has been proposed to distinguish biomagnetite from inorganic and abiotic magnetites (Jimenez-Lopez *et al.*, 2010; Perez-Gonzalez *et al.*, 2010, 2013; Markovski *et al.*, 2017). In this study, the magnetite that precipitated from bioreduction of ferrihydrite and akaganeite shared some of these distinctive features. For example, like previously characterized biomagnetite (Perez-Gonzalez *et al.*, 2010; Markovski *et al.*, 2017), our Raman data show that bioreduced magnetite has a broader band (∼670 cm^−1^) shifted to higher wave numbers relative to synthetic magnetite.

Such a shifted broader band may be due to altered crystal structures, poor crystallinity, or incorporation of other cations (Perez-Gonzalez *et al.*, 2010, 2013). Additionally, Mössbauer data indicate that the magnetite formed was nonstoichiometric, which is typical for biomagnetites, with more oxidized magnetite attributed to associated organic phases (Markovski *et al.*, 2017). Association of organics (*e.g.,* EPS) was evident in our FTIR-ATR data, particularly with *P. delaneyi* grown on ferrihydrite. It is possible that these organics were occluded in the bioreduced magnetite described here as well.

In addition to magnetite, siderite and vivianite were recorded in this study as products of bioreduction by the hyperthermophilic archaea. While these phases have been reported to form from bioreduction of iron oxides by mesophilic (Fredrickson *et al.*, 1998, 2003; Zachara *et al.*, 2002; Roh *et al.*, 2006; Kappler *et al.*, 2014; Sánchez-Román *et al.*, 2014; O'Loughlin *et al.*, 2015, 2019; Markovski *et al.*, 2017) and thermophilic bacteria (Zhang *et al.*, 1997), there has previously been no direct evidence of their formation due to Fe(III) reduction by hyperthermophilic archaea. Vivianite and siderite have been used as proxies for paleoenvironmental conditions, diagenetic evolution of sedimentary sequences, and possible biosignatures (Sapota *et al.*, 2006; Vuillemin *et al.*, 2013, 2019; Sánchez-Román *et al.*, 2014, 2015).

Vivianite plays a major role in the geochemical cycling of P and Fe (Sapota *et al.*, 2006) in reducing environments, particularly in the absence of sulfide (Manning *et al.*, 1991; Sánchez-Román *et al.*, 2015; Rothe *et al.*, 2016). It occurs naturally in both aquatic and terrestrial environments, including marine and freshwater sediments, and hydrothermal deposits (Rothe *et al.*, 2016). Carbonate minerals on Earth are typically considered biogenic, linked to the activity of dissimilatory iron-reducing microbes, and play a critical role in carbon cycling (Dupraz *et al.*, 2009; Sánchez-Román *et al.*, 2015). Siderite, relative to vivianite, is abundantly found in ancient rocks compared with modern environments (Ohmoto *et al.*, 2004; Kholodov and Butuzova, 2008). Both minerals are major components of martian meteorites and the surface of Mars (Valley *et al.*, 1997; Dyar *et al.*, 2014).

This study confirmed that the combined use of reflectance (VNIR, FTIR-ATR), Raman, and Mössbauer spectroscopies is effective in identifying and scrutinizing biogenicity of bioreduced minerals of hyperthermophilic Fe(III) reduction. These techniques were chosen because they can provide complementary and contextual information about nanophase iron oxide minerals and because they represent techniques and tools used in remote sensing of planetary surfaces. Each individual method has its merits and limitations, but all of them collectively showed biogenic mineral transformation of Fe(III) (oxyhydr)oxides during iron respiration relative to heated and unheated abiotic controls.

Fe^3+^ and Fe^2+^ crystal field transitions as well as sample color changes were successfully recorded in VNIR data. VNIR data also show that spectral slopes in two selected regions, 0.475–0.550 and 0.86–1.3 μm, could be used as effective indicators of oxidation state in the minerals examined. While Fe(III) minerals had the most positive slopes, minerals containing Fe(II) showed less positive, neutral, or negative slopes in both regions. Use of spectral slopes in discrete windows in the VNIR has been proposed previously to assess iron oxidation state (Dyar *et al.*, 2018, 2019). Vibrational spectroscopy data from both Raman and FTIR-ATR provided direct evidence for minerals (iron oxide, carbonate, phosphate, and sulfate phases) and organics (cell-associated features), making them especially effective at capturing bioreduction processes.

Raman data were collected by microspectroscopy on selective mineral grains, which made targeted identification of minor phases, such as vivianite and siderite possible when appropriate mineral grains could be found in the samples analyzed. Raman was less effective at capturing organic contributions compared with FTIR-ATR, perhaps due to the choice of laser (532 nm) and grain specificity of the technique. Future studies will need to comparatively examine these products by using other lasers, particularly a 248.6 nm deep ultraviolet laser, more suited for the analysis of organic molecules.

Mössbauer data are best for identifying redox state but limited in interpreting phases with overlapping features and those with contributions <5–10% spectral area. Contextual interpretation with other techniques was necessary to effectively interpret mineral assemblages. Overlapped distributions in the Mössbauer spectra of complex mixtures, particularly with nanophase iron oxides, are common (Dyar *et al.*, 2009). Therefore, phase identification based on fitted parameters was not always reliable or even possible. We recognized this explicitly in our data and emphasized where phase identification was impractical because varied parameters could produce similar fits.

Additionally, many sample spectra showed broadening effects, which likely were due to interparticle interactions, disordered surface domains, and a wide range of particle sizes. Such broadening effects have also been described previously for bioreduction products (Markovski *et al.*, 2017). It is also possible that they are more biologically relevant and warrant further scrutiny among other studies examining mineralogy of microbial processes.

It is imperative to stress that identifications of spectral features identified in this study alone are insufficient in ascribing biogenicity. These features will need to be contextualized with geochemical parameters of an environment, as well as with other targeted metabolic biomarkers of microbial metabolism (Hays *et al.*, 2017). However, when used to identify regions of interest and/or in context of such information, these spectral signatures can provide valuable supporting information in the difficult task of identifying extinct and extant life. One specific and relevant metabolic biosignature that can be coupled with spectral features discussed here are pterin-bearing molecules, which are unique to iron metabolisms (Floyd *et al.*, 2018).

These molecules have been recently shown to be detectable when using another spacecraft relevant technique—thermochemolysis gas chromatography–mass spectrometry in both cultured isolates as well as modern and ancient sedimentary rocks. Pterin-containing proteins are also used for Fe(III) reduction in *P. delaneyi* (Kashyap and Holden, 2021). Therefore, if found in combination with mineral signatures described here would more convincingly suggest the presence of iron cycling microbial metabolisms on planetary surfaces.

## Supplementary Material

Supplemental data

## Appendices

Supplementary Information is included in Appendix A. Mössbauer data are available online at https://mossbauer.mtholyoke.edu/group/Biogeochemical+Mineralogical+By-Products All other spectral data are available in Appendix B as well as online at http://nemo.mtholyoke.edu

## Abbreviations Used

br: broad
cp: cell-associated phosphate
dc: decreased
dp: deep(er)
EPS: extracellular polymeric substances
FTIR-ATR: Fourier-transform infrared attenuated total reflectance
gp: growth medium associated phosphate
ms: missing
neg: negative
neut: neutral
p: Fe^2+^ or mixed Fe^2+^−Fe^3+^ phosphate mineral
pos: positive
sh: sharp
SHERLOC: Scanning Habitable Environments with Raman and Luminescence for Organics and Chemicals
un: unchanged
VNIR: visible–near-infrared
XRD: X-ray diffraction

## Notes

Associate Editor: Nita Sahai
