# Supplementary material for: Spectral Detection of Nanophase Iron Minerals Produced by Fe(III)-Reducing Hyperthermophilic Crenarchaea
Source: Astrobiology. 2022 Dec 30;23(1):43–59. doi: 10.1089/ast.2022.0042 (PMC9810357; doi:10.1089/ast.2022.0042)
Supplement: Supplemental data [file Suppl_Information.pdf]

Supplementary Information

Spectral detection of nanophase iron minerals produced by Fe(III)-reducing hyperthermophilic crenarchaea

Srishti Kashyap, Elizabeth C. Sklute, Peng Wang, Thomas J. Tague, Jr., M. Darby Dyar, James F. Holden

This file contains:

Detailed description of growth media
Detailed description of spectral data acquisiton and analyses
Additional VNIR analysis
VNIR spectral slope analysis
Additional FTIR-ATR analysis
Additional Raman analysis
Additional Mössbauer analysis
Geochemical Modeling
5 supplementary figures
2 tables

Growth media composition

Pyrodictium delaneyi Su06^T was grown in modified DSM 981 medium (Kashefi *et al*., 2002; Kashyap *et al*., 2018). The base medium was composed of the following (per liter): 19 g of NaCl, 9 g of $MgCl_2 \cdot 6H_2O$, 0.3 g of $CaCl_2 \cdot 2H_2O$, 0.5 g of KCl, 0.42 g of KH_2PO_4, 0.05 g of NaBr, 0.02 g of $SrCl_2 \cdot 6H_2O$, 0.15 g of $MgSO_4 \cdot 7H_2O$, 0.1 g of $(NH_4)_2SO_4$, 2.5 g of $NaHCO_3$, 0.2 g yeast extract (Difco, vitamin fortified), 10 ml of DSM Medium 141 trace element solution, 10 ml of DSM 141 vitamin solution and either 100 mmol of ferrihydrite, akaganéite or lepidocrocite. The medium was pH balanced to 6.80 ± 0.05 (room temperature) and 1.3 mM $FeCl_2 \cdot 4H_2O$ and 0.5 mM cysteine-HCl were added prior to inoculation. Incubations were conducted in 160 mL serum bottles containing 50 mL of growth medium sealed with butyl rubber stoppers, degassed and flushed with H_2:CO_2 (80%:20% ratio). Bottles were inoculated using a late-logarithmic growth phase culture that had been grown and transferred at least three times successively on the electron acceptor for that experiment. After inoculation, bottles were over-pressurized with 2 atm of H_2:CO_2 (80%:20% ratio) and incubated at 90°C in a forced-air incubator.

Pyrobaculum islandicum GEO3^T was grown in modified medium that has been described previously (Selig and Schönheit, 1994; Kashyap *et al*., 2018). The growth medium was composed of the following (per liter): 0.33 g of $MgSO_4 \cdot 7H_2O$, 0.33 g of KCl, 0.33 g of NH_4Cl, 0.33 g of $CaCl_2 \cdot 2H_2O$, 0.05 g of KH_2PO_4, 1 mL of 1000× S&S mineral solution, containing (per liter): 2.1 g of $FeSO_4 \cdot 7H_2O$, 12.5 mL of 25% HCl, 30 mg of H_3BO_3, 100 mg of $MnCl_2 \cdot 4H_2O$, 190 mg of $CoCl_2 \cdot 6H_2O$, 24 mg of $NiCl_2 \cdot 6H_2O$, 2 mg of $CuCl_2 \cdot 2H_2O$, 144 mg of $ZnSO_4 \cdot 7H_2O$, and 36 mg of $Na_2MoO_4 \cdot 2H_2O$; 10 mL DSM Medium 141 vitamin solution, 0.2 g yeast extract (Difco), 0.5 g casein hydrolysate, 100 µL of 10 mM $Na_2WO_4 \cdot 2H_2O$ and 1 mM potassium phosphate buffer. Either 100 mmol of ferrihydrite, akaganéite, or lepidocrocite were used as terminal electron acceptors. The pH was balanced to 6.80 ± 0.05 (room temperature). Prior to inoculation, 0.5 mM cysteine-HCl was added to reduce the media, and 1.3 mM $FeCl_2 \cdot 4H_2O$ was added to initiate reduction. Incubations were conducted in 160 mL serum bottles containing 50 mL of growth medium sealed with butyl rubber stoppers, degassed and flushed with N_2. After inoculation, bottles were over-pressurized with 2 atm of N_2 and incubated at 95°C in a forced-air incubator.

Spectral data acquisition and analysis methods

VNIR spectroscopy was performed using an Analytical Spectral Devices (ASD) Fieldspec 4 Max spectrometer with incidence and emission angles set to 30° and 0°, respectively. Spectral data were collected on anoxically dried samples placed in aluminum sample cups (4 mm diameter × 1 mm depth) that were painted matte black. Three spectra of 100 1s integrations were averaged for each sample. Prior to each sample, a dark current and a white reflectance spectrum were collected; white reflectance was set using an ASD Spectralon calibration standard. Peak positions were determined after continuum removal and based on local minima and maxima using the OPUS 7.7 software package (Bruker Optics, Inc.). Samples were examined against reference iron oxides taken from Sklute *et al.* (2018), vivianite (DD-315A) collected in this study from M.D. Dyar's mineral collection, beraunite (C1JB743) acquired by J.L. Bishop with the NASA RELAB facility at Brown University, and siderite (HS271.3B) taken from the USGS Spectral Library (Kokaly *et al*., 2017).

FTIR-ATR spectroscopy used a Bruker Vertex 70 FTIR equipped with a diamond ATR accessory, an ultra-wide range beam splitter (6000-30 cm^{-1}), and a RT-DLaTGS detector.

Spectral data were collected as an average of 128 scans with spectral resolution set at 4 cm^{-1} using Norton Beer strong apodization function. Background spectra were collected at regular intervals to compensate for changing atmospheric and instrumental conditions. For analysis, continuum removal was performed using a rubberband baseline removal algorithm (Wartewig, 2003) and absorption positions determined using local minima in the OPUS 7.7 software package. For broad bands, peak positions were identified by Gaussian and Lorentzian curve fitting algorithms in MagicPlot v.2.9 (MagicPlot Systems, LLC). Multiple peaks were fit iteratively by varying the parameters of the fit function to minimize the residual sum of squares. All spectral data were compared with reference iron (oxyhydr)oxides taken from Sklute *et al.* (2018), iron phosphates, and iron carbonates from M.D. Dyar's mineral collection examined as part of this study to identify mineral transformation products.

Raman spectroscopy was performed using a Bruker Senterra II Raman microscope with a 532 nm excitation laser and a 20× objective at Bruker Optics. Spectral data were collected using either 0.25 mW or 2.5 mW laser power depending on the sensitivity of the sample to phase transformation. Each spectrum was collected using 30-120 2-4s integrations. Multiple individual mineral grains/regions (3-10) per sample were chosen for data acquisition. Darker grains were preferentially analyzed because these likely represented mineral transformations. Spectral data were baseline-corrected with an Asymmetric Least Squares (ALS) algorithm (Eilers and Boelens, 2005) and min-max normalized prior to spectral fitting for feature identification. They were smoothed using a Savitzky-Golay algorithm for visual representation, but no features were assigned based on smoothed data. Band positions were identified using Gaussian and Lorentzian curve fitting algorithms in MagicPlot v.2.9. Multiple peaks were fit iteratively by varying the parameters of the fit function to minimize the residual sum of squares. Phase identifications were confirmed by comparing samples to reference spectra for various iron minerals collected either as part of this study or obtained from the RRUFF database (Lafuente *et al.*, 2015).

Mössbauer spectroscopy was performed using a WEB Research Co. (now SEE Co.) model WT302 spectrometer equipped with a source of ~40 mCi ^{57}Co in Rh and a Janis closed cycle He cooling system (Edina, MN). Spectral data for each sample were acquired over a ± 4 mm s^{-1} velocity range at 295 K and over a ± 10 mm s^{-1} velocity range at 220 K, 150 K, 80 K, and 4 K. They were acquired in 1024 channels and acquisition times varied from 4 h to 5 days depending on the iron content of the samples. All data were corrected for the fraction of the baseline due to Compton scattering of 122 keV gamma rays by electrons inside the detector and non-linearity via interpolation to a linear velocity scale using the 25 □m α-Fe foil calibration spectrum collected at room temperature. The Mössbauer data were fitted using either Mex_disd or Mex_field (De Grave and Van Alboom, 1991). Both programs solve the full hyperfine interaction Hamiltonian for multiple distributions and minimize the chi-squared deviation between the fitted and experimental spectrum using center shift, quadrupole shift, full width at half maximum, and distribution area as adjustable parameters. Mex_field specifically uses Lorentzian-shaped sextets for each distribution but allows for a broadening effect in magnetically ordering samples (Dormann *et al.*, 1985). Mex_disd uses hyperfine field distributions of line-shape independent sextets (Vandenberghe *et al.*, 1994).

Additional VNIR spectroscopy results

The ferrihydrite used in this study displayed a VIS maximum at 0.801 μm, one Fe^{3+} spin-forbidden crystal field transition for $^6A_1 \rightarrow {}^4T_1$ at 0.966 μm, and hydration associated absorption features at 1.403 μm and 1.933 μm. Abiotic ferrihydrite heated controls for both organisms, but

especially for *P. delaneyi*, were significantly muted in overall reflectance and depth of spectral features relative to the ferrihydrite reference spectrum. This decrease in spectral contrast was apparent even in the unheated control for *P. delaneyi* but not for *P. islandicum*, suggesting a possibly strong effect of marine relative freshwater growth medium on ferrihydrite. Despite the decrease in overall reflectance and spectral contrast, all abiotic unheated and heated controls showed the expected VIS maximum feature for ferrihydrite (0.801 µm), although this position was slightly shifted compared to the reference ferrihydrite. Additionally, all abiotic controls except the unheated control for *P. islandicum* were missing the characteristic inflection at 0.966 µm and sharp OH absorption at 1.4 µm typical for ferrihydrite (Scheinost *et al.*, 1998; Sklute *et al.*, 2018). In the unheated abiotic control for *P. islandicum*, the OH absorption was shifted towards higher wavelengths ~1.43 µm, similar to that seen for hematite.

The lepidocrocite used in this study displayed four Fe^{3+} spin-forbidden crystal field transitions at 0.414 µm ($^6A_1 \rightarrow ^4E$), 0.482 µm ($2(^6A_1 \rightarrow ^4T_1)$), 0.726 µm ($^6A_1 \rightarrow ^4T_2$), and 0.978 µm ($^6A_1 \rightarrow ^4T_1$), as well as two local VIS maxima at 0.623 µm and 0.794 µm (Sklute *et al.*, 2018). A similar decrease in spectral contrast relative to the lepidocrocite reference spectrum was noted in the heated abiotic control for *P. delaneyi*. In this sample, positions of the two VIS maxima, as well as the $^6A_1 \rightarrow ^4T_1$ transition, were shifted to 0.593 µm, 0.802 µm and 0.966 µm, respectively, relative to the lepidocrocite spectrum. These features were unchanged in the heated abiotic control for *P. islandicum*, and the unheated abiotic control for both *P. delaneyi* and *P. islandicum.* This suggests that when lepidocrocite was the terminal electron acceptor, heated marine but not freshwater media, abiotically primed the starting mineral for transformation.

The akaganéite used in this study showed five Fe^{3+} spin-forbidden crystal field absorptions at 0.387 µm, 0.428 µm, 0.512 µm ($2(^6A_1 \rightarrow ^4T_1)$), 0.650 µm, and 0.970 µm ($^6A_1 \rightarrow ^4T_1$), a VIS maximum at 0.728 µm, and hydration bands at 1.456 µm, 1.966 µm, 2.324 µm, and 2.452 µm (Sklute *et al.*, 2018). The heated abiotic control for *P. delaneyi* and *P. islandicum* also showed a decline in spectral contrast compared to the unheated abiotic controls and the akaganéite reference. Both samples retained the positions of the $2(^6A_1 \rightarrow ^4T_1)$ absorption at ~0.5 µm; however, the $^6A_1 \rightarrow ^4T_1$ transition typically at 0.97 µm was shifted to shorter wavelengths. Additionally, only the heated abiotic control for *P. islandicum*, but not *P. delaneyi*, lost the characteristic sharpness of the VIS maximum at ~0.728 µm for akaganéite. These differences indicate that structural changes in the starting mineral were growth medium specific and affected by heat in separate ways. Neither the typical absorptions nor the VIS maximum for akaganéite were affected in the unheated abiotic controls for either organism.

VNIR slope analysis

Spectral slopes in the 0.475-0.550 µm and 0.86-1.3 µm (Fig. S1, Table 1) ranges are often associated with Fe^{3+} and Fe^{2+} electronic transitions and are distinctive based on mineral and redox state (Dyar *et al.*, 2018; Dyar *et al.*, 2019). Here, slopes among sample spectra were useful in distinguishing not only redox states, but also the extents of transformation, highlighting subtle differences for the two organisms Positive slopes typically corresponded with Fe^{3+} phases, while negative or neutral slopes with Fe^{2+} or mixed Fe^{3+}- Fe^{2+} phases.

In the 0.475-0.550 µm region, bioreduced ferrihydrite for *P. delaneyi* had a distinctively negative slope, similar to magnetite, while bioreduced ferrihydrite for *P. islandicum* had an intermediate slope between magnetite and ferrihydrite (Fig. S1A). In contrast, all abiotic controls in this region had positive slopes as expected for Fe^{3+} phases. These subtle differences in the bioreduced spectra were consistent with the extent of ferrihydrite reduced by each organism

(Kashyap *et al.*, 2018), with *P. delaneyi* reducing more than *P. islandicum.* Such distinctions were not as apparent in the 0.86-1.3 μm region, because only the unheated control for *P. islandicum* showed a positive spectral slope similar to ferrihydrite (Fig. S1D). All other spectra showed neutral or negative slopes similar to those of magnetite or maghemite, making it difficult to distinguish bioreduced and abiotic spectra using this region alone.

Comparison of spectral slopes in the 0.475-0.550 μm (Fig. S1B) and 0.86-1.3 μm (Fig. S1E) regions for lepidocrocite transformations confirmed subtle differences in these spectra. Only the *P. delaneyi* bioreduced spectrum showed a drastic decrease in slope in the 0.475-0.550 μm region, which appeared similar to ferrous phases, particularly siderite (Fig. S1B). Bioreduced lepidocrocite for *P. islandicum* showed an intermediate slope, indicating less phase transformation, compared to *P. delaneyi*. In contrast, all the heated, unheated abiotic controls, and the lepidocrocite reference spectrum showed sharp positive slopes in this region. Slopes in the 0.86-1.3 μm (Fig. S1E) region in the bioreduced spectra displayed a similar trend. They exhibited a sharp decline becoming neutral, similar to siderite, indicating the formation of a ferrous phase for both organisms. This region also displayed a less positive and more neutral slope, suggesting a minor phase transformation or coating by a dark phase in the heated abiotic control for *P. delaneyi*. All the other heated and unheated abiotic controls showed sharp positive slopes, consistent with the lepidocrocite reference spectrum.

Spectral slopes in the 0.475-0.550 μm (Fig. S1C) and 0.86-1.3 μm (Fig. S1F) regions for akaganéite transformations suggested that the bioreduced spectrum for *P. delaneyi* had the most distinctive Fe^{2+} contributions, similar to magnetite or maghemite. Bioreduced akaganéite for *P. islandicum* had an intermediate or neutral slope, that was indistinct from the heated abiotic control spectrum, particularly in the 0.86-1.3 μm region. The slope for the *P. delaneyi* heated abiotic control in this region was less positive than the unheated abiotic controls and the akaganéite reference, but not negative or neutral as noted in the bioreduced spectrum (Fig. S1F). This suggests that akaganéite, like lepidocrocite, was abiotically primed for reduction in the presence of heated growth medium.

Additional FTIR-ATR spectroscopy results

For the ferrihydrite samples, heated and unheated abiotic controls for both organisms showed subtle changes maintaining similar absorption positions but presenting different band shapes and relative contributions. These changes were particularly prominent in the 500-750 cm^{-1} region (Fig. 2A). For this region, ferrihydrite can be distinguished from other iron oxides by absorption features at 682 cm^{-1}, 630 cm^{-1}, and 588 cm^{-1} in decreasing order of relative contribution. For *P. delaneyi,* both abiotic controls displayed a deeper absorption at ~588 cm^{-1} relative to the absorptions at ~680 cm^{-1} and ~630 cm^{-1} for ferrihydrite. The heated abiotic control spectrum lacked the 630 cm^{-1} feature altogether and displayed the ~680 cm^{-1} feature but shifted to higher wavenumbers. The unheated abiotic control for *P. islandicum* showed change in band shapes and absorption positions for this region similar to those noted in the unheated abiotic control for *P. delaneyi.* In contrast, the heated abiotic control for *P. islandicum* showed a slightly deeper absorption at 588 cm^{-1}, lacked the 630 cm^{-1} feature, but maintained the position of the absorption at ~680 cm^{-1} relative to ferrihydrite. Below 750 cm^{-1}, the heated abiotic control for *P. islandicum* showed many similarities in band position, but not band shape and contribution to bioreduced ferrihydrite for *P. islandicum*. This distinction was crucial for identifying the more subtle biogenic mineral transformations that occurred in the case of *P. islandicum.*

The features in the phosphate region (900-1200 cm^{-1}) were expanded to lower wavenumbers in the bioreduced spectrum for *P. delaneyi* compared to the unheated and heated abiotic control spectra. This shifted position was also accompanied with a change in shape of these features, making them more consistent with iron-phosphate minerals like beraunite ($Fe^{2+}Fe^{3+}_5(OH)_5(PO_4)_4{\cdot}4H_2O$), vivianite ($Fe_3(PO_4)_2{\cdot}8H_2O$), or chalcosiderite ($CuFe^{3+}_6(PO_4)_4(OH)_8{\cdot}4H_2O$). However, all iron phosphate minerals also have a phosphate bending mode at ~450 cm^{-1} (Frost *et al.*, 2002; Frost *et al.*, 2014), which was absent in the bioreduced ferrihydrite spectrum for *P. delaneyi*. Therefore, it is possible that either 1) a different iron phosphate mineral formed, 2) no iron phosphate mineral formed but rather phosphate ions in solution changed between biotic and abiotic conditions, or 3) some of these distinctive features were cell associated and cell specific. Bioreduced ferrihydrite for *P. islandicum* also showed a similar shift and expansion of these phosphate vibrations towards lower wavenumbers, but unlike *P. delaneyi*, this was limited to only one of the three absorptions in this region at ~950 cm^{-1}. While sulfate vibrations are also found in this region, they typically extend above 1150 cm^{-1} (Lane, 2007; Sklute *et al.*, 2015), making them unlikely contributions in sample spectra. In all three spectra, the ~1465 cm^{-1} feature for HCO_3^- sorbed on ferrihydrite was absent, and the ~1360 cm^{-1} feature was shifted to higher wavenumbers.

For the lepidocrocite samples, the unheated and heated abiotic controls showed shifted feature positions and contributions relative to the lepidocrocite reference, suggesting the possible formation of new phases. For instance, diagnostic lepidocrocite features, associated with OH vibrations at ~1150 cm^{-1}, ~750 cm^{-1}, and ~460 cm^{-1} had shifted positions. Additionally, features at ~635 cm^{-1} and 585 cm^{-1} changed in depth and position, with the former peak increased in depth, and latter shifted to ~600 cm^{-1}. A new shoulder also appeared at ~168 cm^{-1} in these abiotic control spectra relative to the lepidocrocite reference. However, the most striking change was the broadened feature that spanned from ~850-1260 cm^{-1} in both abiotic control spectra for *P. delaneyi*. Its position was consistent with symmetric and antisymmetric phosphate stretching vibrations, either as a mineral or solution or sorbed phase. The absence of features at ~780 cm^{-1} or at ~500 cm^{-1} suggested that chalcosiderite, vivianite, beraunite or phosphosiderite ($FePO_4{\cdot}2H_2O$) did not form. However, it is plausible that a different phosphate mineral phase not considered here formed in the abiotic conditions. These changes were distinct from those in the bioreduced spectrum for *P. delaneyi*, in that this entire phosphate region band envelope disappeared, and instead, carbonate features appeared. Such distinct spectral differences between experimental conditions suggest that any Fe^{2+} (and/or Fe^{3+}) iron involved in the formation of phosphate phases in the abiotic conditions, got consumed in forming siderite upon lepidocrocite bioreduction by *P. delaneyi*.

Typical akaganéite features were also affected in all three conditions for *P. islandicum* as well as the heated abiotic control and bioreduced spectrum for *P. delaneyi*. The most significant changes were noted in the absorption features of a combined asymmetric band at ~630 cm^{-1} and ~686 cm^{-1}. The 630 cm^{-1} feature is attributed to a δ_{OH} out-of-plane bend, and the 686 cm^{-1} represents the combination of an OH-bending and a ν_4 sulfate vibration (Murad and Bishop, 2000; Bishop *et al.*, 2015). Additionally, contributions at 650 cm^{-1} for this asymmetric band represent OH…Cl in akaganéite (Bishop *et al.*, 2015). In all but the unheated abiotic control for *P. delaneyi*, mid-peak contributions at ~650 cm^{-1} decreased in area and intensity, with the lowest intensities in both the bioreduced spectra.

Additional Raman spectroscopy results

Growth media differences and heat without cells caused subtle mineral changes and band position shifts towards lower wavenumbers for the starting oxides (Table 3).

Minerals such as ferrihydrite, maghemite, and magnetite have highly overlapped features in the 650-730 cm^{-1} region and show only subtle differences among each other. Ferrihydrite shows a major peak at ~710 cm^{-1} (Fig. 3A) (Hanesch, 2009; Das *et al.*, 2013; Sklute *et al.*, 2018), maghemite shows two peaks at 665 cm^{-1} and 730 cm^{-1} (Hanesch, 2009), and magnetite shows a single peak at 670 cm^{-1} (Hanesch, 2009; Sklute *et al.*, 2018). However, because all three minerals display an asymmetric peak in this region, bioreduced and abiotic ferrihydrite samples were directly compared to nanophase reference standards based on Gaussian fitting. Within this range, curve fitting illustrated that ferrihydrite had the highest wavenumber features (695 cm^{-1}, 735 cm^{-1}), magnetite had the lowest wavenumber features (668 cm^{-1}, 702 cm^{-1}) and maghemite displayed one low and one high wavenumber feature (670 cm^{-1}, 720 cm^{-1}).

With lepidocrocite, the *P. delaneyi* unheated and heated abiotic controls displayed all the typical bands for lepidocrocite, although these were slightly shifted to lower wavenumbers (Table 3). In addition, only in the heated control, the feature at 1056 cm^{-1}, expected for lepidocrocite, was shifted to 1073 cm^{-1}, indicating possible contributions from the symmetric stretching mode of a carbonate phase, similar to an iron hydroxycarbonate, such as chukanovite (Saheb *et al.*, 2011). Other modes for chukanovite appeared to be lacking, so it is possible that this feature is instead due to aqueous carbonate sorbed on mineral oxides. For the *P. islandicum* lepidocrocite samples, the feature at 382 cm^{-1} was found at 377 cm^{-1} in the unheated control, 375 cm^{-1} in the heated control, and 373 cm^{-1} in bioreduced lepidocrocite. Similarly, the band at 1057 cm^{-1} was present at 1053 cm^{-1} in the unheated control, 1046 cm^{-1} in the heated control, and 1036 cm^{-1} in bioreduced lepidocrocite (Table 3, Fig. 3B). The latter band lies in the region (~950-1100 cm^{-1}) where symmetric and antisymmetric stretching modes of PO_4^{3-} ions are expected (Frost *et al.*, 2002; Litasov and Podgornykh, 2017). Within this range, features in ferrous phosphates typically are shifted towards lower wavenumbers and features in ferric or mixed ferrous-ferric phosphates towards higher wavenumbers (Fig. S2). Furthermore, the *P. islandicum* bioreduced spectrum also showed a slight increase in intensity at 310 cm^{-1} (Fig. 3B). This feature was likely due to a FeO stretching vibration overlapped with the lattice mode of PO_4^{3-} (Frost *et al.*, 2002; Litasov and Podgornykh, 2017; Sklute *et al.*, 2018).

P. delaneyi and *P. islandicum* heated and unheated abiotic controls with akaganéite displayed all the expected major bands for akaganéite at 318 cm^{-1}, 389 cm^{-1}, 545 cm^{-1}, and 725 cm^{-1}, and additionally when fitted showed a minor feature in the 1040-1060 cm^{-1} range, possibly from abiotic phosphate in the growth medium (Fig. 3C, Table 3). While the magnetite reference (12 nm) reported in this study showed an increase in the relative band intensity at 330 cm^{-1}, Dar and Shivashankar (2013) noted that a 30 nm-synthesized magnetite could also have the same position as our reference for the T_{2g} mode. However, polycrystalline films of 40 nm and 200 nm magnetite synthesized by Jubb and Allen (2010) showed this feature at 310 cm^{-1}, as did the magnetite (grain size unknown) reported by Hanesch (2009). The magnetite that formed from akaganéite bioreduction in this study had a broader range of larger grained spheres (10-250 nm) (Kashyap *et al.*, 2018). It is possible that the position of the T_{2g} mode in magnetite is grain size dependent, with larger grain sizes showing a shift towards lower wavenumbers, but further work would be needed to confirm this hypothesis.

Additional Mössbauer spectroscopy results

Synthetic ferrihydrite Mössbauer spectra were fit with two doublets (Fig. 4A) or three sextets (Fig. S3D) of Lorentzian lineshape as they allow for more consistent fits and clearer data interpretation. With *P. delaneyi*, the unheated, abiotic control spectra were largely consistent with the reference ferrihydrite (Fig. 4A, Fig S3A), but showed a small ferrous contribution (1% spectral area, within the error of the technique) resolvable at >80K. At 4K, the spectrum resembled that of the reference. The heated, abiotic control appeared indistinguishable from the reference ferrihydrite at all temperatures (Fig. 4A). For *P. islandicum*, the unheated, abiotic control appeared similar to the reference ferrihydrite, other than a slight increase in magnetic ordering temperature (apparent at 80K; Fig. 4A, Fig. S3). The heated abiotic control similarly magnetically ordered by 80K (Fig. 4A, Fig. S3) but also showed changes in the combined ferrihydrite peak shape at 4K, specifically in the separation of sextets in the lowest velocity peak (Fig. 3SD). Additionally, minor hematite (3% spectral area) was resolvable at all temperatures.

Reference Mössbauer spectra for lepidocrocite were fit with two Lorentzian doublets ≥80K (Fig. 4B; Fig. S4) and with two sextets at 4K. Lepidocrocite doublets displayed moderately increasing CS (0.38-0.49 mm/s) and minimally increasing QS (0.52/0.62 – 0.54/0.66 mm/s). Lepidocrocite sextets had QS values near zero and fields below 460 kOe (Fig. S4D). With *P. delaneyi,* Mössbauer spectra for both unheated and heated abiotic controls also showed a small Fe^{2+} phase (3-4% spectral area) (Fig. 4B; Fig. S4). Fit parameters for this phase vary between abiotic controls but the peak is not sufficiently resolved to conclude these changes indicate mineralogical differences. Below 220K, unheated and heated abiotic controls both displayed a small Fe^{3+} sextet (5-8% spectral area) (Fig. 4B, Fig. S4B-D). This phase, which is likely superparamagnetic and overlapped with lepidocrocite at 295K, appeared best resolved at 80K, with CS = 0.48 mm/s, QS = -0.26 mm/s, and hyperfine field = 484 kOe. This is most consistent with nanophase goethite (Fig. 4B). In the *P. delaneyi* bioreduced spectra, one distribution (CS = 1.23 mm/s, QS = 2.56 mm/s) displayed the same parameters as the small Fe^{2+} doublet in the abiotic controls. In addition to Fe^{2+} distributions and lepidocrocite, the bioreduced spectrum also contained a small Fe^{3+} sextet below 220K similar to the one observed in abiotic controls (Fig. 4B, Fig. S4B-D). With *P. islandicum,* Mössbauer spectra and fit parameters of the unheated and heated abiotic controls were consistent with those of reference lepidocrocite (Fig. 4B).

At 295K, reference nanophase akaganéite was fit with two doublets and one disordered sextet (Fig. S5A). While akaganéite is often adequately fit with two doublets (García *et al.*, 2004; Barrero *et al.*, 2006; Klimas *et al.*, 2015), increased H_2O (correlated with decreased Cl^-) has been shown to affect ordering temperature, leading to such a sextet (Chambaere *et al.*, 1978). This distribution is unsurprising in our data since all minerals were stored and used as fluid suspensions. At 220K, three sextets with similar CS but different QS and hyperfine fields were resolvable (Fig. 4C; Fig. S5B-D). The sextet with the most negative QS was associated with the smallest hyperfine field and decreased in area to 8% by 4K. This is consistent with literature reports that conclude this distribution results from temperature effects, not a particular Fe site (Rezel and Genin, 1990). For *P. delaneyi*, the unheated abiotic control was similar to the reference but also contained a small, unresolvable Fe^{2+} doublet (see baseline near 0 mm/s at 220K and 150K) (Fig. 4C; Fig. S5B-C). The heated abiotic control had altered sextet structure at 295K (Fig. S5A), which can be seen at lower temperatures to be due, in part, to Fe^{2+} contributions. This Fe^{2+} doublet is similar to that in the *P. islandicum* heated control at 220K, but

the 150K and 80K fit parameters show this is a different phase (Fig. 4C, Fig. S5C). For *P. islandicum*, the unheated abiotic control appeared similar to reference akaganéite over all temperatures. The heated abiotic control additionally displayed a Fe^{2+} doublet at 220K (~7% spectral area) (Fig. S5B) and also a Fe^{3+} component that persisted far below the splitting temperature for akaganéite. If the Fe^{3+} and Fe^{2+} components are from the same mineral, it could be a mixed valent phosphate or sulfate (Dyar *et al.*, 2013; Dyar *et al.*, 2014). Adsorbed Fe(II) can display parameters similar to those for the Fe^{2+} doublet (ThomasArrigo *et al.*, 2014), but all Fe^{2+} components in this sample magnetically order at 4K, making adsorbed Fe^{2+} an unlikely explanation.

Geochemical modeling: Methods and Results

To predict mineral phases that precipitate from the growth media, components of each growth medium were speciated and analyzed using the REACT module in The Geochemist's Workbench v.10.0 software package (Aqueous Solutions, LLC). For ferrihydrite analysis, Fe^{3+} was swapped with precipitated $Fe(OH)_3$. For lepidocrocite and akaganéite analysis, Fe^{3+} was swapped with goethite. All minerals were allowed to precipitate and dissolve in the model during mixing. C, Fe, and NH_4^+ were decoupled from the reactions but all other reactions were allowed. To account for the hydrogenotrophic conditions for *P. delaneyi*, $H_{2(aq)}$ with a fugacity of 1.92 was swapped for O_2. The module was run at pH 6.8 and 90°C for *P. delaneyi* and pH 6.8 and 95°C for *P. islandicum*. Mineral saturation states based on log Q/K values at equilibrium were used to assess which of the supersaturated minerals were expected to precipitate.

Table S1 and S2 show the mineral saturation states for initial and final conditions of the growth medium for *P. delaneyi* and *P. islandicum*, respectively, with either ferrihydrite, lepidocrocite, or akaganéite as the terminal electron acceptor. Initial conditions were simulated to be indicative of the unheated and heated abiotic controls, and final conditions were simulated to represent an equivalent amount of Fe^{2+} as present in bioreduced conditions.

With ferrihydrite, lepidocrocite, and akaganéite, saturation index values indicated that the *P. delaneyi* growth medium initially was favorable to abiotic precipitation of hydroxyapatite, magnesite, vivianite, hematite, and pyrrhotite (Table S1). Geochemical modeling of final conditions indicated that the growth medium for *P. delaneyi* provided conditions suitable for abiotic precipitation of all these phases, as well as siderite with ferrihydrite, lepidocrocite, and akaganéite as the starting minerals (Table S1). Magnetite and possibly vivianite were identified for ferrihydrite bioreduced by *P. delaneyi* in our dataset. Bioreduced lepidocrocite, on the other hand, resulted in the formation of siderite, some ferrous phosphate, and possibly nanophase goethite, while bioreduced akaganéite showed formation of magnetite, and vivianite. Given that some but not all the identified phases were predicted in the geochemical modeling, it was apparent that metabolic activity and presence of *P. delaneyi* controlled mineral precipitation processes.

For *P. islandicum,* initial conditions with ferrihydrite were favorable for the precipitation of vivianite, hematite, and hydroxyapatite (Table S2). Final conditions with ferrihydrite predicted that only vivianite and hematite of these phases were saturated. With lepidocrocite and akaganéite, only hematite and hydroxyapatite were saturated in the growth medium initially, but whitlockite, hematite and vivianite precipitated under final conditions. Magnetite, which was produced from bioreduction of ferrihydrite by *P. islandicum,* was not predicted in geochemical modeling. However, for lepidocrocite and akaganéite transformations, modeled results matched

identified iron mineral phases, except for hematite, suggesting that abiotic mineral precipitation processes largely controlled phase formation in the case of *P. islandicum.*

REFERENCES

Barrero CA, García KE, Morales AL, *et al*. New analysis of the Mössbauer spectra of akaganeite. *J Phys Condens Matter* 2006;18:6827-6840.

Bishop JL, Murad E, Dyar MD. Akaganéite and schwertmannite: spectral properties and geochemical implications of their possible presence on Mars. *Am Mineral* 2015;100:738-746.

Chambaere D, Govaert A, De Sitter J, *et al*. A Mössbauer investigation of the quadrupole splitting in β-FeOOH. *Solid State Commun* 1978;26:657-659.

Dar MI, Shivashankar SA. Single crystalline magnetite, maghemite, and hematite nanoparticles with rich coercivity. *RSC Adv* 2013;4:4105-4113.

Das S, Hendry MJ, Essilfie-Dughan J. Adsorption of selenate onto ferrihydrite, goethite, and lepidocrocite under neutral pH conditions. *Appl Geochem* 2013;28:185-193.

De Grave E, Van Alboom A. Evaluation of ferrous and ferric Mössbauer fractions. *Phys Chem Minerals* 1991;18:337-342.

Dormann JL, Cui JR, Sella C. Mössbauer studies of Fe_2O_3 antiferromagnetic small particles. *J Appl Phys* 1985;57:4283-4285.

Dyar MD, Breves E, Jawin E, *et al*. Mössbauer parameters of iron in sulfate minerals. *Am Mineral* 2013;98:1943-1965.

Dyar MD, Jawin ER, Breves E, *et al*. Mössbauer parameters of iron in phosphate minerals: Implications for interpretation of Martian data. *Am Mineral* 2014;99:914-942.

Dyar MD, Helbert J, Boucher T, *et al*. Venus surface oxidation and weathering as viewed from orbit with six-window VNIR spectroscopy [abstract 2137]. In 16th Meeting of the Venus Exploration and Analysis Group (VEXAG) Conference Abstracts, Applied Physics Laboratory, Laurel, MD, 2018.

Dyar MD, Helbert J, Maturilli A, *et al*. Interpreting Venus surface spectra from orbit: Insights in rock type and oxidation from laboratory data [abstract P14B-08]. In American Geophysical Union Annual Fall Meeting Conference Abstracts, San Francisco, CA, 2019.

Eilers PHC, Boelens HFM. Baseline correction with asymmetric least squares smoothing. Leiden University Medical Centre Report, 2005.

Frost RL, Martens W, Williams PA, *et al*. Raman and infrared spectroscopic study of the vivianite-group phosphates vivianite, baricite and bobierrite. *Mineral Mag* 2002;66:1063-1073.

Frost RL, López A, Scholz R, *et al*. The molecular structure of the phosphate mineral beraunite $Fe^{2+}Fe_5^{3+}(PO_4)_4(OH)_5 \cdot 4H_2O$: A vibrational spectroscopic study. *Spectrochim Acta A* 2014;128:408-412.

García KE, Morales AL, Barrero CA, *et al*. Magnetic and crystal structure refinement in akaganeite nanoparticle. *Physica B* 2004;354:187-190.

Hanesch, M. Raman spectroscopy of iron oxides and (oxy)hydroxides at low laser power and possible applications in environmental magnetic studies. *Geophys J Int* 2009;177:941-948.

Jubb AM, Allen HC. Vibrational spectroscopic characterization of hematite, maghemite, and magnetite thin films produced by vapor deposition. *ACS Appl. Mater Interfaces* 2010;2:2804-2812.

Kashefi K, Tor JM, Holmes DE, *et al*. *Geoglobus ahangari* gen. nov., sp. nov., a novel hyperthermophilic archaeon capable of oxidizing organic acids and growing autotrophically on hydrogen with Fe(III) serving as the sole electron acceptor. *Int J Syst Evol Microbiol* 2002;52:719-728.

Kashyap S, Sklute EC, Dyar MD, *et al*. Reduction and morphological transformation of synthetic nanophase iron oxide minerals by hyperthermophilic archaea. *Front Microbiol* 2018;9:1550.

Klimas V, Mažeika K, Jasulaitienė V, *et al*. Formation, morphology and composition of F^-- and Cl^--stabilized iron β-oxyhydroxides. *J Fluor Chem* 2015;170:1-9.

Kokaly RF, Clark RN, Swayze GA, *et al*. USGS Spectral Library Version 7. U.S. Geological Survey, Reston, VA, 2017.

Lafuente B, Downs RT, Yang H, *et al*. The power of databases: the RRUFF project. In Highlights in Mineralogical Crystallography. De Gruyter, Berlin, 2015, pp 1-30.

Lane MD. Mid-infrared emission spectroscopy of sulfate and sulfate-bearing minerals. *Am Mineral* 2007;92:1-18.

Litasov KD, Podgornykh NM. Raman spectroscopy of various phosphate minerals and occurrence of tuite in the Elga IIE iron meteorite. *J Raman Spectrosc* 2017;48:1518-1527.

Murad E, Bishop JL. The infrared spectrum of synthetic akaganéite, β-FeOOH. *Am Mineral* 2000;85:716-721.

Rezel D, Genin JMR. The substitution of chloride ions to OH^--ions in the akaganeite beta ferric oxyhydroxide studied by Mössbauer effect. *Hyperfine Interact* 1990;57:2067-2075.

Saheb M, Neff D, Bellot-Gurlet L, *et al*. Raman study of a deuterated iron hydroxycarbonate to assess long-term corrosion mechanisms in anoxic soils. *J Raman Spectrosc* 2011;42:1100-1108.

Scheinost AC, Chavernas A, Barrón V, *et al*. Use and limitations of second-derivative diffuse reflectance spectroscopy in the visible to near-infrared range to identify and quantify Fe oxide minerals in soils. *Clays Clay Miner* 1998;46:528-536.

Selig M, Schönheit P. Oxidation of organic compounds to CO_2 with sulfur or thiosulfate as electron acceptor in the anaerobic hyperthermophilic archaea *Thermoproteus tenax* and *Pyrobaculum islandicum* proceeds via the citric acid cycle. *Arch Microbiol* 1994;162:286-294.

Sklute EC, Jensen HB, Rogers AD, *et al*. Morphological, structural, and spectral characteristics of amorphous iron sulfates. *J Geophys Res Planets* 2015;120:809-830.

Sklute EC, Kashyap S, Dyar MD, *et al*. Spectral and morphological characteristics of synthetic nanophase iron (oxyhydr)oxides. *Phys Chem Minerals* 2018;45:1-26.

ThomasArrigo LK, Mikutta C, Byrne J, *et al*. Iron and arsenic speciation and distribution in organic flocs from streambeds of an arsenic-enriched peatland. *Environ Sci Technol* 2014;48:13218-13228.

Vandenberghe RE, De Grave E, de Bakker PMA. On the methodology of the analysis of Mössbauer spectra. *Hyperfine Interact* 1994;83:29-49.

Wartewig S. IR and Raman spectroscopy: fundamental processing. Wiley, New York, 2003.

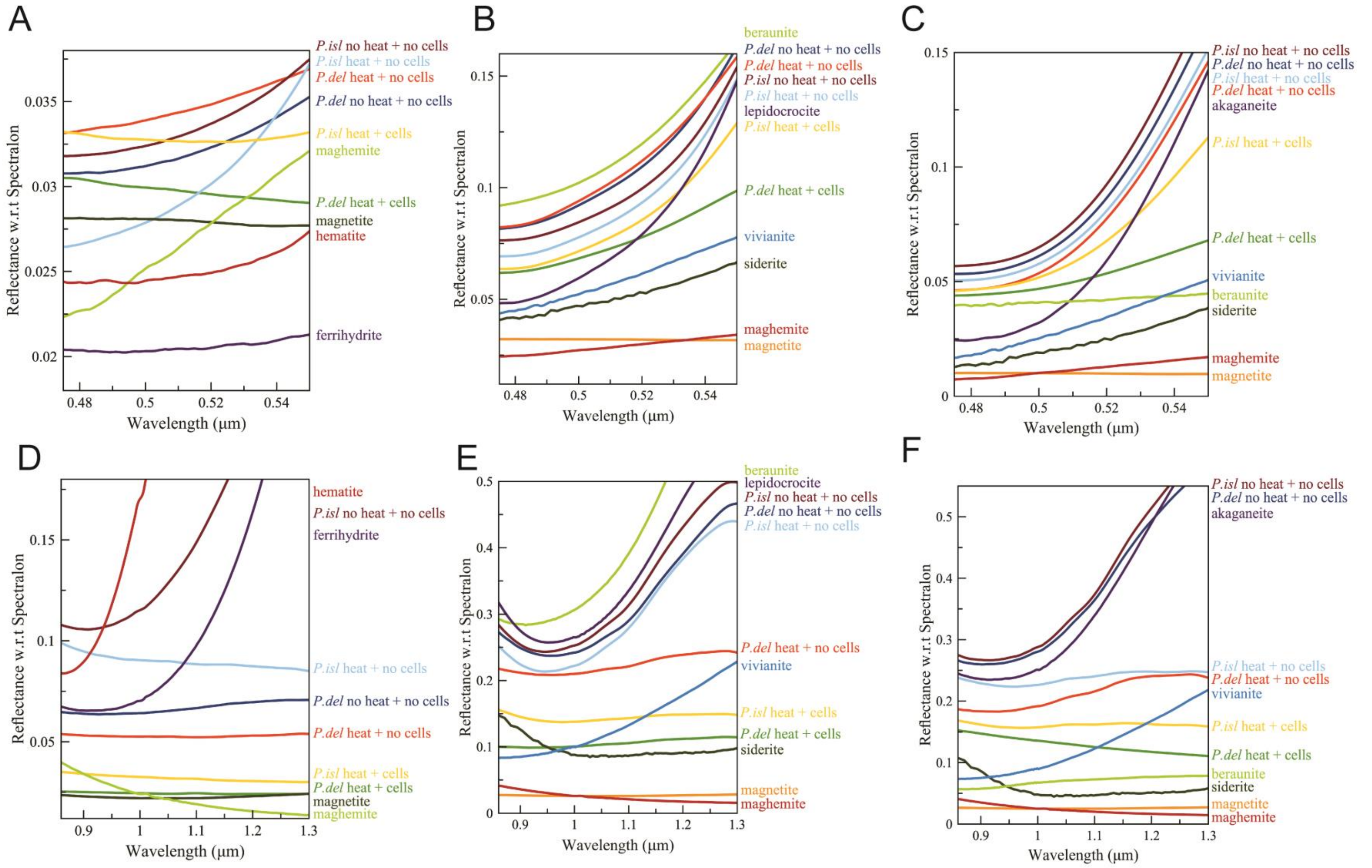

Fig. S1. VNIR spectra of biotic (‘heat + cells’), heated abiotic (‘heat + no cells’) and unheated abiotic (‘no heat + no cells’) controls of *P. delaneyi* and *P. islandicum* grown on ferrihydrite (A,D), lepidocrocite (B,E) and akaganeite (C,F) along with iron mineral reference standards to highlight slopes in the 0.475-0.550 μm (A,B,C) and 0.86-1.3 μm (D,E,F) regions.

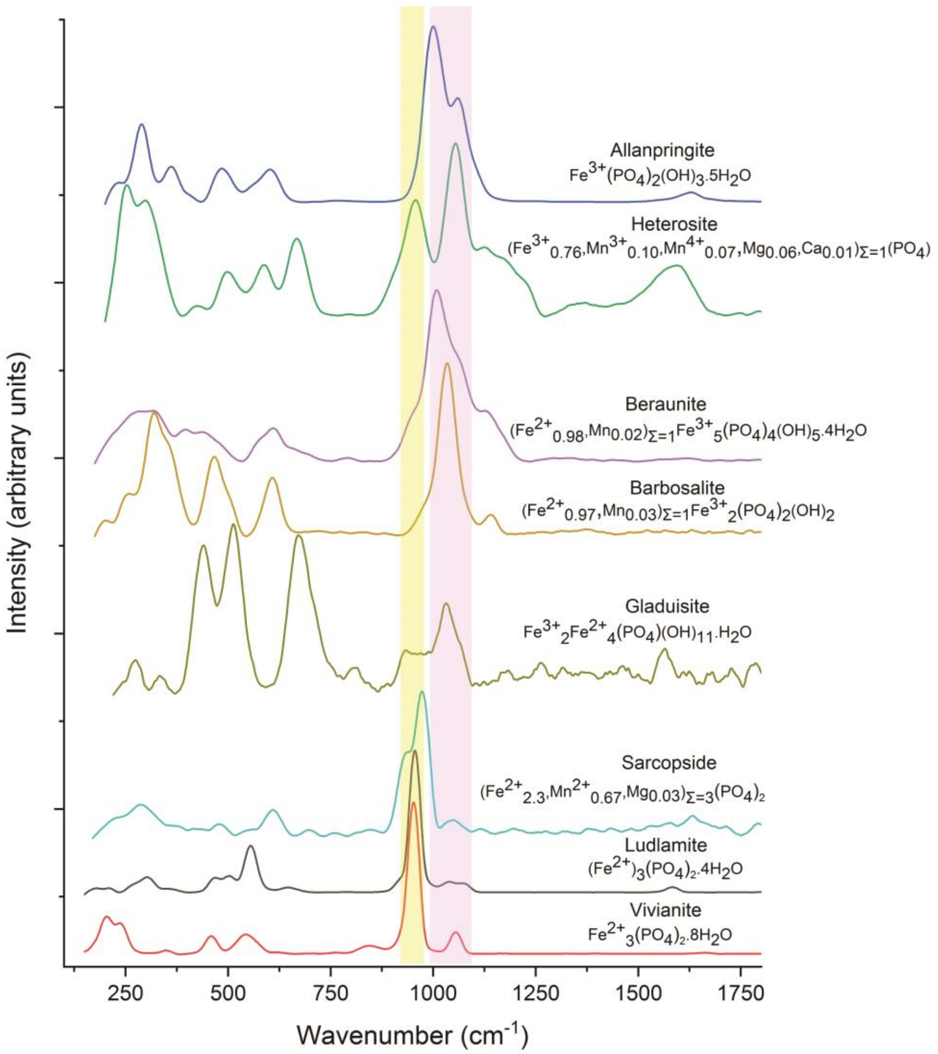

Fig. S2. Raman spectra of iron phosphates obtained from the RRUFF database – vivianite (050076), ludlamite (040152), sarcopside (070553), gladuisite (070432), barbosalite (070358), beraunite (060696), heterosite (070148), allanpringite (080147). Measured chemistry is described where known for each mineral. Yellow shaded region is the narrow range noted for stretching modes of phosphate in Fe^{2+} phosphates and purple shaded region for mixed valent (Fe^{2+}- Fe^{3+}) as well as Fe^{3+} phosphates.

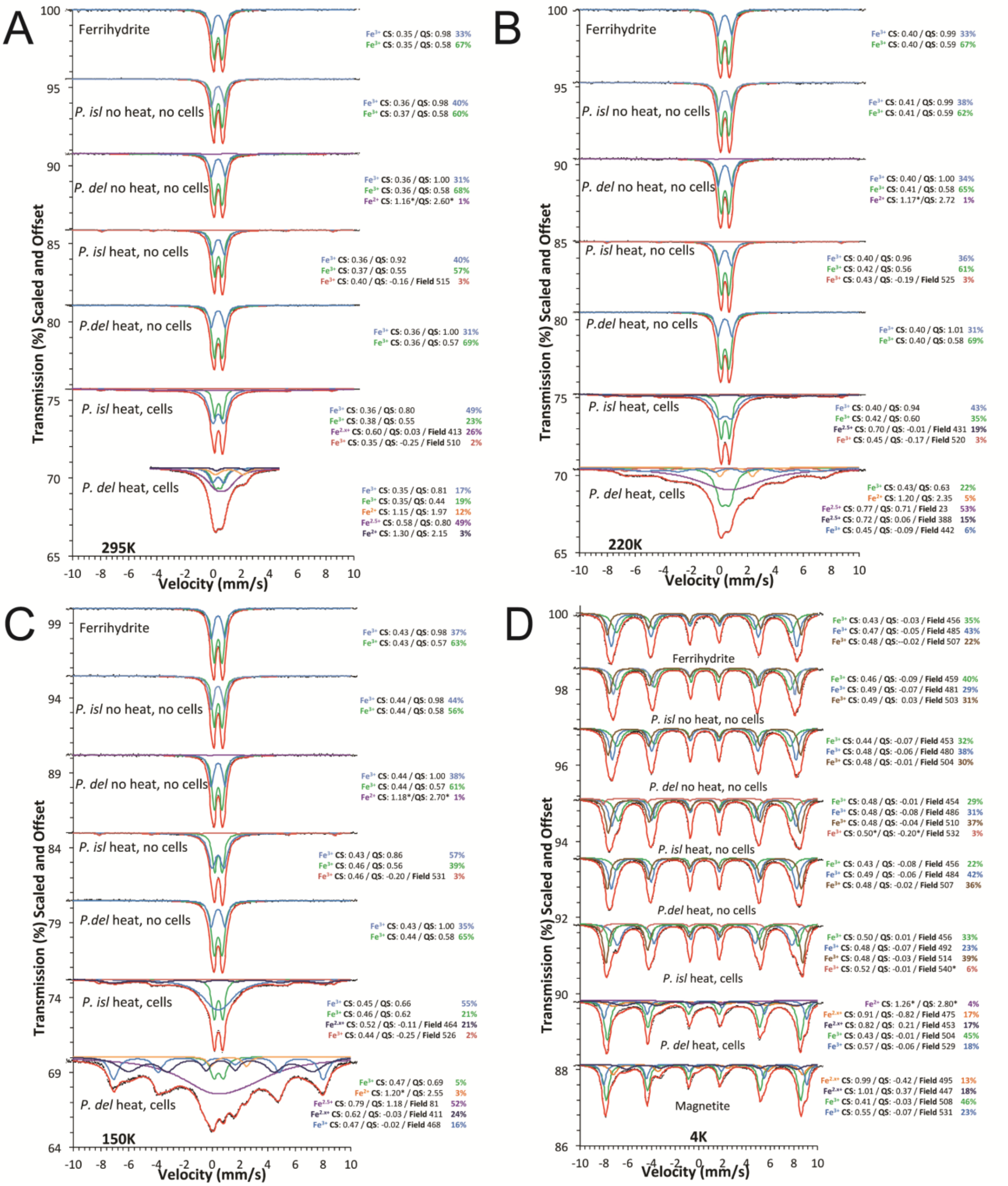

Fig. S3. Mössbauer spectra of *P. delaneyi* (*P. del*) and *P. islandicum* (*P. isl*) grown on ferrihydrite. The raw and fitted spectra and all fitted parameters are shown for the unheated abiotic control ('no heat, no cells'), heated abiotic control ('heat, no cells'), and biotic ('heat, cells') conditions of each organism along with reference ferrihydrite and magnetite (at 4K). Each spectrum is scaled and offset for clarity. The temperature of acquisition is shown at the base of the x axis.

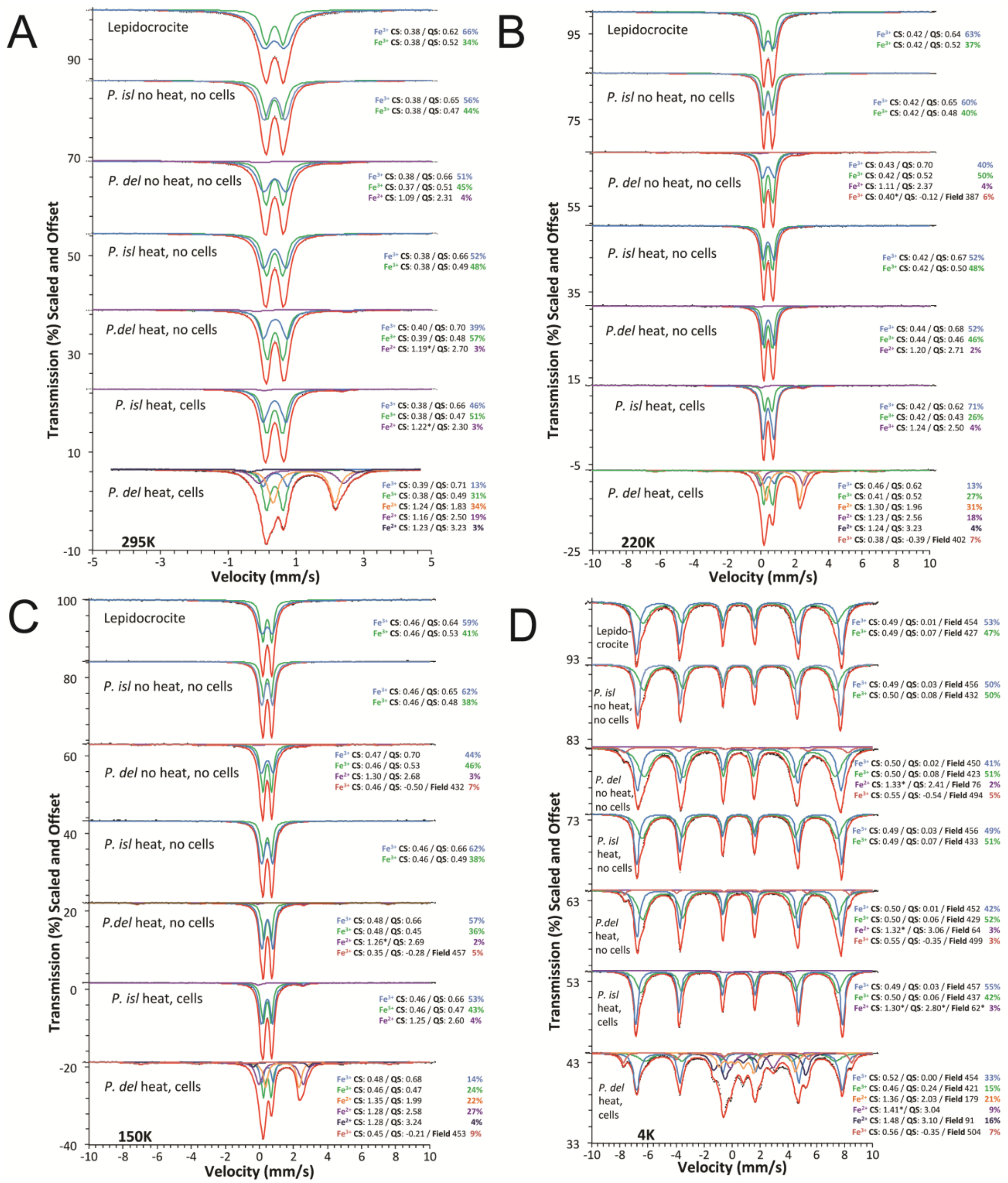

Figure S4. Mössbauer spectra of *P. delaneyi* (*P. del*) and *P. islandicum* (*P. isl*) grown on lepidocrocite. The raw and fitted spectra and all fitted parameters are shown for the unheated abiotic control ('no heat, no cells'), heated abiotic control ('heat, no cells'), and biotic ('heat, cells') conditions of each organism along with reference lepidocrocite. Each spectrum is scaled and offset for clarity. The temperature of acquisition is shown at the base of the x-axis.

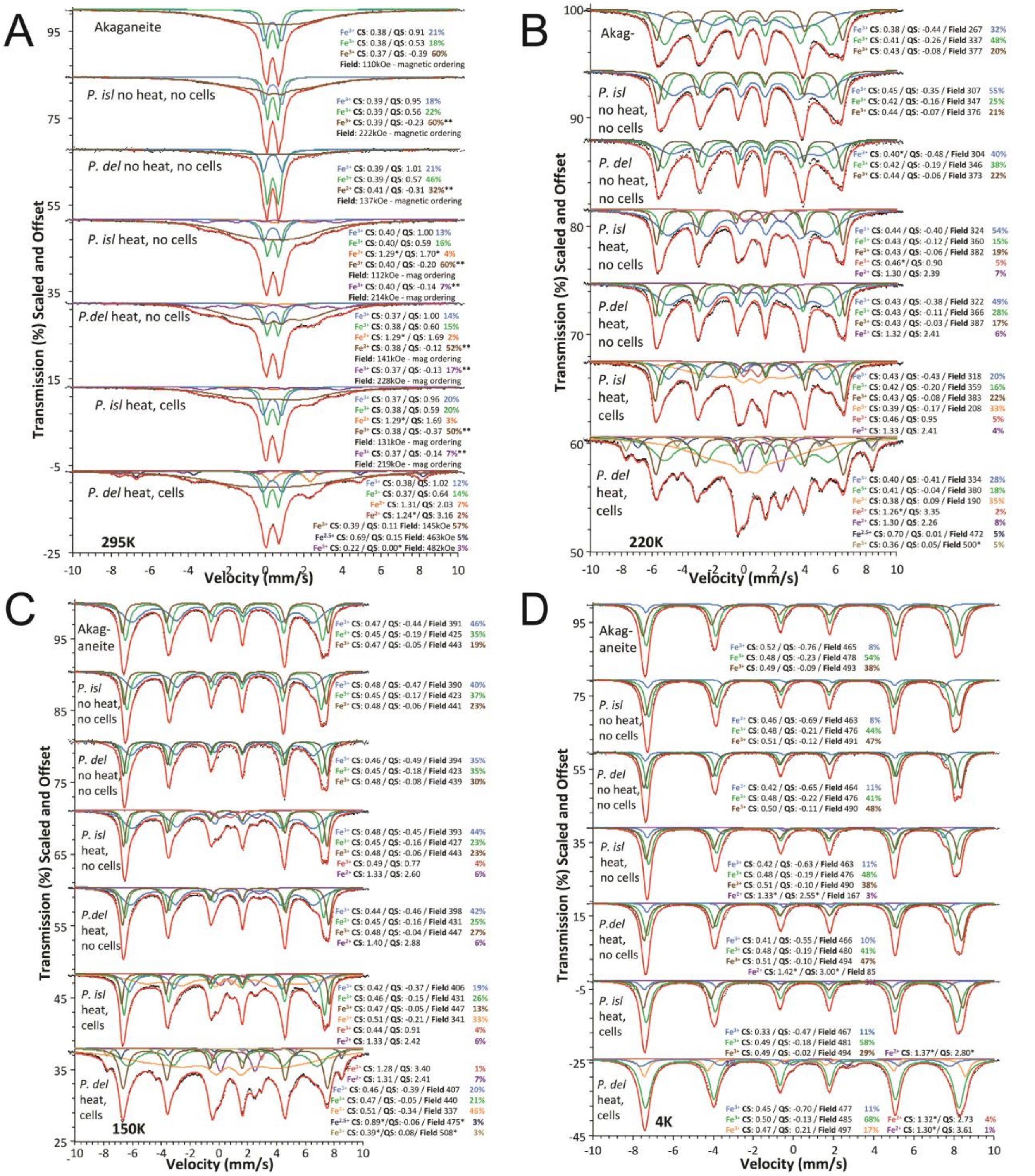

Fig. S5. Mössbauer spectra of *P. delaneyi* (*P. del*) and *P. islandicum* (*P. isl*) grown on akaganeite. The raw and fitted spectra and all fitted parameters are shown for the unheated abiotic control ('no heat, no cells'), heated abiotic control ('heat, no cells'), and biotic ('heat, cells') conditions of each organism along with reference akaganeite. Each spectrum is scaled and offset for clarity. The temperature of acquisition is shown at the base of the x-axis.

Table S1. Mineral saturation states in the *P. delaneyi* marine growth medium using the REACT module in Geochemist's Workbench. Values reported are for equilibrated initial and final conditions.

Ferrihydrite (Initial)		Ferrihydrite (Final)		Lepidocrocite/Akaganeite (Initial)		Lepidocrocite/Akaganeite (Final)	
Mineral	Saturation State log Q/K	Mineral	Saturation State log Q/K	Mineral	Saturation State log Q/K	Mineral	Saturation State log Q/K
Hydroxyapatite	0/sat	Vivianite	0/sat	Hydroxyapatite	0/sat	Vivianite	0/sat
Magnesite	0/sat	Pyrrhotite	0/sat	Magnesite	0/sat	Siderite	0/sat
Vivianite	0/sat	Siderite	0/sat	Vivianite	0/sat	Pyrrhotite	0/sat
Hematite	0/sat	Hydroxyapatite	0/sat	Hematite	0/sat	Hematite	0/sat
Pyrrhotite	0/sat	Hematite	0/sat	Pyrrhotite	0/sat	Magnesite	0/sat
Whitlockite	-0.2576	Whitlockite	-0.3667	Whitlockite	-0.2573	Hydroxyapatite	0/sat
Siderite	-0.4308	Troilite	-0.5317	Siderite	-0.4309	Whitlockite	-0.3760
Strontianite	-0.4807	Goethite	-0.5969	Strontianite	-0.4807	Strontianite	-0.5138
Goethite	-0.5966	Magnesite	-0.6346	Goethite	-0.5966	Troilite	-0.5206
Troilite	-0.6689	Magnetite	-0.8012	Troilite	-0.6693	Goethite	-0.5967
Magnetite	-1.1671	Strontianite	-1.2115	Magnetite	-1.168	Magnetite	-0.7716
Dolomite	-1.1909	Pyrite	-1.3319	Dolomite-ord	-1.1904	Dolomite-ord	-0.7996
Pyrite	-1.2379	Dolomite-ord	-1.4320	Dolomite	-1.1909	Dolomite	-0.8001
FeO(c)	-2.056	Dolomite	-1.4325	Pyrite	-1.2386	Pyrite	-1.3382
$SrHPO_4$(c)	-2.2422	FeO(c)	-1.6902	FeO(c)	-2.057	FeO(c)	-1.6606
Dolomite-dis	-2.3501	Calcite	-2.1042	$SrHPO_4$(c)	-2.2417	Dolomite-dis	-1.9593
Calcite	-2.4972	Aragonite	-2.2644	Dolomite-dis	-2.3501	Calcite	-2.1064
Aragonite	-2.6574	Dolomite-dis	-2.5916	Calcite	-2.4972	Aragonite	-2.2666
Halite	-2.7968	Halite	-2.7648	Aragonite	-2.6574	Halite	-2.7832
				Halite	-2.7953	$SrHPO_4$(c)	-2.9032

*sat=saturated

Table S2. Mineral saturation states in the *P. islandicum* freshwater growth medium using the REACT module in Geochemist's Workbench. Values reported are for equilibrated initial and final conditions.

Ferrihydrite (Initial)		Ferrihydrite (Final)		Lepidocrocite/Akaganeite (Initial)		Lepidocrocite/Akaganeite (Final)	
Mineral	Saturation State log Q/K	Mineral	Saturation State log Q/K	Mineral	Saturation State log Q/K	Mineral	Saturation State log Q/K
Vivianite	0/sat	Vivianite	0/sat	Hematite	0/sat	Whitlockite	0/sat
Hematite	0/sat	Hematite	0/sat	Hydroxyapatite	0/sat	Hematite	0/sat
Hydroxyapatite	0/sat	Goethite	-0.6031	Whitlockite	-0.0416	Vivianite	0/sat
Whitlockite	-0.0237	Celestite	-0.9639	Goethite	-0.6031	Hydroxyapatite	-0.1767
Goethite	-0.6031	Anhydrite	-1.2501	Anhydrite	-2.1634	Goethite	-0.6030
Magnetite	-1.8307	Strengite	-1.6815	Strengite	-2.2669	Celestite	-0.8293
Anhydrite	-1.8744	Gypsum	-1.6918	Gypsum	-2.6052	Anhydrite	-1.1661
Strengite	-2.1775	Whitlockite	-1.7013	Bassanite	-2.8024	Strengite	-1.5281
Gypsum	-2.3162	Bassanite	-1.8891	$CaSO_4.0.5H_2O$	-2.8962	Gypsum	-1.6074
Bassanite	-2.5135	$CaSO_4.0.5H_2O$	-1.9830			Bassanite	-1.8050
$CaSO_4.0.5H_2O$	-2.6073	Magnetite	-2.1615			$CaSO_4.0.5H_2O$	-1.8988
FeO(c)	-2.7057	Hydroxyapatite	-2.9614			Magnetite	-2.2640
						$SrHPO_4$(c)	-2.7667

*sat=saturated
